# Calbindin-D28k deficiency mediates tau-driven hippocampal hyperexcitement and cognitive impairment

**DOI:** 10.1186/s40035-026-00547-3

**Published:** 2026-05-12

**Authors:** Yang Gao, Xiaoqing Tao, Yuying Wang, Yarong Wang, Huan Li, Yang Yu, Mengqi Tu, Yanchao Liu, Jie Zhou, Yuchen Li, Wei Wei, Xiaochuang Wang, Jie Zheng, Yao Zhang, Haibo Xu, Jian-Zhi Wang

**Affiliations:** 1 Department of Radiology, Zhongnan Hospital of Wuhan University, Wuhan University, Wuhan, 430071 China; 2 Department of Pathophysiology, Key Laboratory of Ministry of Education for Neurological Disorders, School of Basic Medicine, Tongji Medical College, Huazhong University of Science and Technology, Wuhan, 430030 China; 3 Hubei Key Laboratory of Cognitive and Affective Disorders, Institutes of Biomedical Sciences, School of Medicine, Jianghan University, Wuhan, 430056 China; 4 Key Laboratory of Ministry of Education for Neurological Disorders, Department of Endocrine, Liyuan Hospital, Tongji Medical College, Huazhong University of Science and Technology, Wuhan, 430077 China; 5 Department of Clinical Research and Translational Medicine, The Third Affiliated Hospital of Zhengzhou University, Zhengzhou, 450052 China; 6 Department of Rehabilitation Medicine, General Hospital of the Yangtze River Shipping, Wuhan, 430015 China; 7 Neuroscience Research Institute and Department of Neurobiology, School of Basic Medical Sciences, Peking University. Key Laboratory for Neuroscience, Ministry of Education, National Health Commission, Peking University, Beijing, 100083 China; 8 Hubei Provincial Engineering Research Center of Multimodal Medical Imaging Technology and Clinical Application, Wuhan, 430071 China; 9 Co-Innovation Center of Neuroregeneration, Nantong University, Nantong, 226000 China

**Keywords:** Alzheimer's disease, Temporal lobe epilepsy, Tau hyperphosphorylation, Calbindin-D28k, Cognitive impairment, Calcium homeostasis

## Abstract

**Background:**

Medial temporal lobe hyperexcitation or seizures originating from the hippocampus are frequently observed in Alzheimer's disease (AD) patients, contributing to accelerated cognitive decline. As the hippocampus is an early vulnerable area of tau pathology, the mechanisms by which abnormal tau aggregation promotes temporal lobe epilepsy (TLE) remain poorly understood.

**Methods:**

We investigated the role of AD-like hippocampal tau aggregation in neuronal hyperexcitation using transgenic (Tg) tau-driven mice. Effects of tau aggregation on intracellular calcium dynamics were assessed by calcium imaging. Neuronal/network hyperexcitability and seizure susceptibility were evaluated through patch-clamp electrophysiology, ^18^F-FDG PET/CT, and optogenetic induction. A tetracycline-controlled (Tet-on) system in Tg hTau368 mice enabled spatiotemporal induction of tau pathology to investigate its interactions with calbindin-D28k (CB) and synaptic proteins. Adeno-associated virus (AAV)-mediated CB supplementation in hippocampal CA1 and dentate gyrus (DG) excitatory neurons was performed to correct hyperexcitability and cognitive deficits. Finally, the relationship between CB and disease progress was analyzed using an AD public database.

**Results:**

Tau accumulation in the hippocampal CA1/DG CaMKII-positive excitatory neurons reduced CB expression with disruption of calcium homeostasis. This dysregulation increased neuronal excitability, diminished synaptic protein levels, and increased seizure susceptibility and cognitive impairment. AAV-driven CB restoration in CA1/DG neurons attenuated both hyperexcitability and cognitive deficits. In the brains of AD patients, reduced CB expression was associated with cognitive deterioration and advanced disease stages.

**Conclusions:**

Tau aggregation drives calcium dysregulation and hippocampal neuronal hyperexcitation through reducing CB expression. These results establish a potential mechanistic link between tauopathy and TLE pathogenesis in AD, providing evidence for CB as a promising therapeutic target for mitigating seizure risk and related cognitive decline in AD.

**Supplementary Information:**

The online version contains supplementary material available at 10.1186/s40035-026-00547-3.

## Introduction

Alzheimer's disease (AD) is the most common and devastating neurodegenerative disease, with a lack of effective prevention or approved therapies yet [1–3]. A striking clinical feature of AD is the higher prevalence of temporal lobe epilepsy (TLE) and 42%–64% of AD patients exhibit overt epileptic seizures or subclinical epileptic activity [4–7]. Notably, early-onset AD patients (aged 50–60 years) show an 87-fold increased incidence of seizures compared to age-matched healthy individuals [8]. Intracranial recordings reveal that silent hippocampal seizures and spikes emerge during the preclinical phase of AD, preceding overt cognitive symptoms [9]. Human studies demonstrated that increased neuronal network and brain activity start as early as a decade before the onset of disease [10–12]. Hippocampal excitability follows an inverted U-shaped trajectory during AD progression, with hyperexcitability in early stages transitioning to hypoexcitability in later phases [4, 13]. Critically, seizures in AD patients accelerate cognitive decline and increase the mortality risk due to trauma from sudden loss of consciousness [14, 15].

Extracellular amyloid beta (Aβ) plaque deposition and intraneuronal neurofibrillary tangles (NFTs) in the brain are two characteristic pathological hallmarks of AD. However, they demonstrate distinct spatiotemporal progression patterns. The Aβ deposition predominates in the medial parietal and frontal cortices, whereas tau pathology initially accumulates within the entorhinal-hippocampal network at prodromal stages [16, 17]. PET imaging in patients with early clinical stages of AD who developed TLE revealed highly asymmetric tau deposition in the epileptogenic hemisphere, whereas the asymmetry of amyloid deposition was less pronounced [7]. The entorhinal-hippocampal network contains key excitatory pathways: the entorhinal cortex (EC) efferents directly innervate the dentate gyrus (DG), where granule cells extend mossy fiber axons to CA3. CA3 neurons subsequently relay signals to CA1 via Schaffer collaterals, while parallel EC projections establish direct monosynaptic connections with CA1 pyramidal neurons. Notably, the DG–CA1–EC–DG loop has been identified as one of the principal circuits involved in seizure generation and propagation of TLE [18]. Functional neuroimaging revealed enhanced BOLD (blood-oxygen-level-dependent) activation in the hippocampal-temporal circuits during memory tasks in early AD [11, 12]. Glutamatergic neurotransmission, mediated by CA1 pyramidal neurons and DG granule cells, critically regulates learning and memory processes [18, 19]. Of particular significance, the CA1 pyramidal layer demonstrates selective vulnerability to early tau deposition [20, 21]. Nevertheless, the precise mechanisms through which tau pathology disrupts hippocampal excitatory-inhibitory balance remain to be elucidated.

Calcium homeostasis is of utmost significance in maintaining the excitatory-inhibitory balance within neural circuits, recently regarded as an important target for AD treatment [22, 23]. Among the various calcium regulators, Calbindin-D28k (CB), a calcium-binding protein, is highly expressed in hippocampal CA1 and DG neurons [24–26]. Functionally, CB serves as a key regulator of intracellular free calcium ion concentrations, actively participating in the modulation of intracellular calcium homeostasis, synaptic plasticity, and cognitive processes [25, 27]. A decline in CB expression has been consistently associated with cognitive impairment in patients suffering from AD and TLE [27]. Evidence from animal models further supports this relationship: knockdown of CB in the excitatory neurons of hippocampal CA1 and DG leads to significant impairments in the spatial memory of mice [26]. Clinical observations have also revealed reduced CB expression in the hippocampal CA1 and DG regions of AD and TLE patients [27]. However, it is unknown whether replenishing CB in the excitatory neurons of hippocampal CA1 and DG could alleviate neuronal over-excitation and mitigate cognitive impairment. Addressing this question could potentially uncover new therapeutic strategies for treating AD and TLE.

This study aimed to elucidate how AD-like tau aggregation in the early vulnerable hippocampal circuitry contributes to local network hyperexcitability and susceptibility to TLE. To address the relationship between tau aggregation and hippocampal hyperexcitability, we employed a newly created tauopathy Tg hTau368 mice to investigate tau aggregation feature and calcium dysregulation. Neuronal hyperexcitability and seizure susceptibility were evaluated using patch-clamp electrophysiology, ^18^F-FDG PET/CT and optogenetic activation. To support the premise that CB may serve as a potential therapeutic target, we used a tetracycline-inducible (Tet-on) system in Tg hTau368 mice to achieve spatiotemporally controlled induction of tau pathology and to delineate its interactions with CB and synaptic proteins. In parallel, adeno-associated virus (AAV)-mediated CB restoration in hippocampal CA1/DG excitatory neurons was performed to evaluate whether enhancing CB levels could ameliorate neuronal hyperexcitability and cognitive deficits. Finally, AD-related public databases were analyzed to corroborate the clinical relevance of CB by examining associations between CB expression and cognitive outcomes.

## Methods

### Animals and human brain tissue

All mice were housed in groups of three to four per cage, under a 12 h light/dark cycle at 23–25 °C. Food and water were provided ad libitum. Doxycycline hyclate (Dox, Beyotime, Shanghai, China) was dissolved in drinking water (2 mg/L) and administered ad libitum for Tg hTau368 mice. Equal numbers of male and female mice were randomly assigned to groups. All experiments and data analyses were conducted by experimenters blind to the groupings. All animal experiments were conducted in accordance with relevant ethical regulations for animal testing and research, and were approved by institutional guidelines and the Animal Care and Use Committee of Tongji Medical College, Huazhong University of Science and Technology. Human brain tissue sections were obtained from the Department of Forensic Medicine, Zunyi Medical University, Guiyang, China. The use of these samples was approved by the Ethics Committee of Guizhou Medical University (Approval No. 2023091).

### Tg hTau368 and PR5 mice

The Tg hTau368 mice were generated jointly by our laboratory and Nanjing Biomedical Research Institute of Nanjing University. Briefly, the human *MAPT* gene encoding the hTau368 fragment (2N4R tau) was inserted downstream the tetracycline-responsive element (TRE) promoter, to assemble a Tet-on system with a second module expressing the reverse tetracycline-controlled transactivator (rtTA) under the control of the neuron-specific Eno2 promoter [28]. In the presence of Dox (Dox-on), rtTA binds to the TRE to initiate hTau368 expression. In the absence of doxycycline (Dox-off), hTau368 expression is not expressed, as rtTA cannot bind to TRE [28].

The PR5 mice overexpress the longest human tau isoform (2N4R tau) with a P301L mutation on the C57BL/6 background under the control of the murine Thy1.2 promoter. This leads to the formation of sarcosyl-insoluble, 15-nm-wide tau filaments. These filaments are comparable to those observed in patients with frontotemporal dementia with parkinsonism linked to chromosome 17 carrying the P301L mutation [29, 30].

### Stereotaxic viral injection

Mice were anesthetized by 2% isoflurane (RWD Life Science, Shenzhen, China) and secured in a stereotaxic frame (RWD Life Science). The scalp was surgically incised to expose the skull, and residual soft tissue on the skull surface was meticulously removed using sterile cotton swabs. The coordinate origin was defined as the intersection of the bilateral tangents at the bregma. The skull was leveled by aligning the anterior and posterior bregma, ensuring a height discrepancy of less than 0.03 mm between the left and right sides. The target injection site was identified based on stereotaxic coordinates and marked on the skull. A small craniotomy was performed at the marked location using a precision cranial drill. Viral solution was delivered at a rate of 100 nL/min using a microinjection system and retained for 5 min post-injection (Additional file 1: Table S1).

### Kainic acid (KA)-induced seizure model and in vivo electrophysiological recording

KA, an analog of glutamate, was used to induce TLE by activating kainate receptors, leading to seizure activity, neuronal damage, and chronic spontaneous epilepsy. To explore the role of CA1 and DG in seizures, KA (500 nL, 0.5 μg/μL, dissolved in saline) was stereotaxically injected into the hippocampal CA1 (− 1.0 mm AP, + 1.8 mm ML, − 1.5 mm DV) and DG (− 1.0 mm AP, + 1.8 mm ML, − 2.1 mm DV), respectively. An 8-channel electrode was implanted 0.1 mm above the injection sites to record electrophysiological signals when mice were awake. Local field potentials were recorded using Plexon OmniPlex System (Plexon, Hong Kong, China). Data were stored for offline analysis with 16-bit format, visualized in NeuroExplore. Amplitude and power spectral density (PSD) of LFPs were analyzed by NeuroExplorer (Plexon) in default parameters: shift, 0.5 s; number of frequency values, 8192; normalization, log of PSD; show frequency, 0–150 Hz.

### Optogenetic stimulation and seizures induction

Optogenetic stimulation was performed according to our previous report [31]. Briefly, pAAV-CaMKIIα-ChR2 (H134R)-mCherry (BrainVTA, Wuhan, China) was stereotaxically injected into the right dorsal hippocampal CA1 (− 1.0 mm AP, + 1.8 mm ML, − 1.1 mm DV, 500 nL) and DG (− 1.0 mm AP, + 1.8 mm ML, − 2.1 mm DV, 500 nL), or the right ventral hippocampal CA1 (− 3.2 mm AP, + 3.2 mm ML, − 4.1 mm DV, 500 nL) and DG (− 3.2 mm AP, + 3.2 mm ML, − 4.8 mm DV, 500 nL). Following viral injection, a fiber optic cannula (200 μm core, numerical aperture = 0.37, RWD Life Science) was implanted at 0.4 mm above CA1. An 8-channel electrode was implanted into the ipsilateral M1 (+ 1.5 mm AP, + 1.5 mm ML, –1.5 mm DV) for electrophysiological recording. Anchoring screws (25-μm diameter) were fixed into the skull near the injection site. The cannula and screws were secured with dental cement (RWD Life Science). Four weeks post-surgery, mice were anesthetized with isoflurane, and the cannula was connected to a fiber optic patch cable and the laser source. Seizures were stimulated using laser parameters of 472 nm blue light, 20 Hz frequency, 10% duty cycle, and pulsed waveform. The actual output power of optical fiber outlet was 2.8 mW/mm^2^.

Seizure latency and severity were assessed using a modified Racine scale [32]: Stage 1, facial twitching and chewing; Stage 2, chewing and head nodding; Stage 3, unilateral forelimb clonus; Stage 4, bilateral forelimb clonus with rearing; Stage 5, bilateral clonus, rearing, and falling; Stage 6, wild jumping. Stages 1–3 were classified as focal seizures (FS), and stages 4–6 as generalized seizures (GS). The latency to stage 4 (GS) was recorded as the primary metric in our study. Upon reaching stage 4, stimulation continued for 15 s before cessation. The maximum seizure stage was recorded, and mice were monitored until normal behavior resumed. The fiber optic cable was removed under anesthesia, and mice were returned to their cages. Seizures were induced once daily, with GS latency and maximum seizure stage recorded over consecutive days (Supplementary Video 1).

### Small animal PET/CT imaging

Brain ^18^F-FDG PET/CT imaging was conducted using a small animal PET/CT scanner (Novel Medical, Beijing, China) at Union Hospital, Wuhan. Mice were fasted for 12 h, weighed, and injected with 3–4 MBq of ^18^F-FDG via the tail vein. Anesthesia was induced and maintained with 2% isoflurane. After a 45-min uptake period, whole-body PET/CT scans were performed with the following parameters: energy window 350–650 keV, timing window 1.2 ns, spatial resolution 1.5 mm, and scan duration 300 s. Acquired DICOM images were analyzed using the PMOD 4.0 software. A standardized mouse brain template was used to segment the brain into 19 regions of interest (ROIs), and the average ^18^F-FDG uptake intensity for each ROI was quantified as standardized uptake value normalized to body weight.

### Monitoring of mouse energy metabolism and activity

The Oxymax CLAMS system (Columbus Instruments, Columbus, OH) was employed to monitor the 24-h energy metabolism and locomotor activity of mice. Prior to the experiment, mice were acclimated to the laboratory environment for 24 h, separately housed in cages, ensuring an adequate supply of food and water. Subsequently, the oxygen consumption (VO₂), carbon dioxide production (VCO₂), respiratory quotient (RQ), and spontaneous activity counts of each mouse were recorded over a 24-h period (light cycle: 7:00–19:00). The energy expenditure was calculated as follows: Total energy expenditure = (3.815 + 1.232 × RQ) × VO₂. This system utilizes infrared photocell technology to enable three-dimensional monitoring of animal movements. Mouse locomotion interrupts the infrared beam, which generates a count for spontaneous movement.

### Calcium imaging recording

Four weeks prior to imaging, pAAV-CaMKIIα-GCaMP6f (500 nL) was injected into the right hippocampal CA1 region (− 1.0 mm AP, + 1.8 mm ML, − 1.5 mm DV) in the Dox and Veh groups (*n* = 4 per group). Mice were anesthetized with 1% sodium pentobarbital (intraperitoneal injection) and decapitated. Brains were quickly removed, trimmed, and affixed to a vibratome stage using cyanoacrylate glue. Coronal slices (300 μm thickness) were cut in ice-cold slicing solution and transferred to oxygenated (95% O₂, 5% CO_2_) artificial cerebrospinal fluid at 35 °C for 30 min, followed by 1-h incubation at room temperature before imaging. Brain slices were observed under a Zeiss (Germany) LSM 510 confocal imaging system with a 40 × water immersion objective (Achroplan, 0.8 numerical aperture; Zeiss). ROIs corresponded to the visually identifiable CA1 pyramidal cell layer with green fluorescence signal (GCaMP6f). Neuronal depolarization was induced by perfusing the slices with 30 mM KCl. Fluorescence signals were excited at 488 nm and recorded within the 505–550 nm emission range. Images were acquired every 5 s (0.2 Hz) and analyzed using ImageJ software. Calcium signals were calculated using the following formula: Δ*F*/*F* = [(*F*1 − *B*1) − (*F*0 − *B*0)]/(*F*0 − *B*0), where *F*1and *F*0 represent fluorescence intensities during and before KCl stimulation, and *B*1 and *B*0 represent background signals. Data were normalized, with the baseline signal set to 0%.

### In vitro patch-clamp electrophysiological recording

All experiments were conducted using solutions prepared as follows: Cutting Solution containing choline chloride (110 mM), KCl (2.5 mM), NaH₂PO₄ (1.25 mM), NaHCO₃ (26.0 mM), CaCl₂ (0.5 mM), MgSO₄ (7 mM), D-glucose (10 mM), Na-ascorbate (11.6 mM), Na-pyruvate (3.1 mM), and atropine sulfate (0.01 mM); Recording Solution containing NaCl (119 mM), KCl (2.5 mM), NaH_2_PO_4_ (1.25 mM), NaHCO_3_ (26 mM), CaCl₂ (2.5 mM), MgCl₂ (1.3 mM), and *D*-glucose (10 mM); K⁺ Intracellular Solution containing K-gluconate (128 mM), KCl (17.5 mM), Na₂ATP (5 mM), MgCl₂ (1 mM), EGTA (0.2 mM), and HEPES (10 mM), adjusted to pH 7.4 and osmolarity of 290–300 mOsm.

Two-month-old homozygous Tg hTau368 mice were randomly divided into three groups (Veh + eGFP, Dox + eGFP, Dox + CB). Viral vectors (AAV-CaMKIIα-eGFP or AAV-CaMKIIα-Calb1-eGFP) were bilaterally injected into hippocampal CA1 and DG 1.5 months prior to recording. Brain slices were prepared, transferred to a perfusion chamber, and stabilized under a microscope, where green-fluorescent CA1 pyramidal neurons in optimal physiological condition were selected for recording. For spontaneous excitatory postsynaptic currents (sEPSCs), a glass electrode (3–5 MΩ) filled with K⁺ intracellular solution was used to clamp neurons at − 70 mV after gigaseal formation and membrane rupture; sEPSCs were recorded for 120 s in voltage-clamp mode. Action potentials (APs) were recorded in current-clamp mode using the same electrode, applying step-current stimuli (-20–140 pA, 20 pA increments, 10 s intervals) and counting APs within 600 ms windows. Data were acquired via a Multiclamp 700B amplifie and Digidata 1440 A digitizer (sampling at 10 kHz, low-pass filtered at 2 kHz), and analysis was performed using the Clampfit 10.6 software (Molecular Devices, CA) for frequency, amplitude, and AP quantification.

### Western blotting

The hippocampus was separated on ice and homogenized in a customized RIPA lysis buffer containing 50 mM Tris (pH 7.4), 150 mM NaCl, 1% Triton X-100, 1% sodium deoxycholate, and 0.1% SDS (Beyotime, Shanghai, China). To prevent protein degradation and maintain phosphorylation states, a protease and phosphatase inhibitor cocktail (Thermo Scientific, Waltham, MA) was added at a ratio of 10 μL per mg of tissue. Protein concentrations within the RIPA-soluble and RIPA-insoluble lysates (centrifuged at 12,000 × *g* for 20 min, Beyotime, Shanghai, China) were determined [28]. The proteins were separated by SDS-PAGE and electrophoretically transferred onto nitrocellulose membranes (Merck Millipore, Darmstadt, Germany). The membranes were then blocked with 5% bovine serum albumin (BSA) to prevent non-specific antibody binding. Subsequently, they were incubated sequentially with primary and secondary antibodies (Table 1). Protein bands were visualized using an enhanced chemiluminescence substrate system (Santa Cruz Biotech, Dallas, TX). The membranes were imaged with an Odyssey Imaging System (LI-COR Biosciences, Lincoln, NE), and band intensities were quantified using the Image J. β-Actin was used as a loading control to ensure consistent protein loading across samples.

**Table 1:** Antibodies used in this study

Antibody	Type	Specificity	Species	Application	Source/reference
Tau368	Mono	Tau 1–368 N	M	IMF/WB	Jointly developed with AtaGenix [28]
Tau5	Mono	Tau (a.a.210–230)	M	WB	Abcam (ab80579)
HT7	Mono	Tau (a.a.159–163)	M	WB	ThermoFisher (MN10000)
Anti-pT205	Poly	p-tau (T205)	R	IMF/WB	SAB (11108)
AT8	Mono	p-tau (S202/T205)	M	IMF/IMH/WB	ThermoScientific (MN1020)
PSD-95	Poly	PSD-95	R	WB	SAB (41365)
SYN-1	Mono	Synapsin1	M	WB	CST (D12G5)
β-actin	Mono	β-actin	M	WB	SAB (21800)
Anti-Calbindin	Mono	Calbindin-D28k	R	IMH/WB	Abcam (ab108404)
CaMKII	Mono	CaMKII	R	IMF	Cell Signaling (3362)
GAD67	Mono	GAD67	M	IMF	Sigma (MAB5406)
Anti-Parvalbumin	Mono	Parvalbumin	M	IMF	Sigma (SAB4200545)
GFAP	Mono	GFAP	M	IMF	CST (3670)
Iba1	Mono	Iba1	G	IMF	SAB (ab5076)

Antibody dilutions: 1:200 for immunostaining; 1:1000 for Western blotting (WB)

Abbreviations: Mono, monoclonal; Poly, polyclonal; M, mouse; R, rabbit; G, goat; IMH, immunohistochemistry; IMF, immunofluorescence

### Immunohistochemistry and immunofluorescence staining

Mice were anesthetized with 2% isoflurane (RWD Life Science), transcardially perfused with 0.9% NaCl for 5 min to clear the blood, followed by perfusion with 4% paraformaldehyde in PBS for 1 day. The brains were treated with 25% sucrose for 1 day and then transferred to 30% sucrose for an additional day. Using a cryostat microtome (Leica, Wetzlar, Germany), the brains were cut into 30-μm-thick sections.

For immunohistochemistry, the free-floating sections were first immersed in 3% H_2_O_2_ in anhydrous methanol for 30 min to quench endogenous peroxidases. Non-specific binding sites were blocked with BSA for 30 min at room temperature. The brain slices were then incubated overnight at 4 °C with primary antibodies. The immunoreactions were developed using a DAB staining kit (ZSGB-BIO, Beijing, China). Images of the stained sections were captured at 20 × magnification using an automatic slice scanning system (Olympus, Tokyo, Japan) and analyzed with ImageJ software. The areas of different brain regions were measured to assess the staining distribution.

For immunofluorescence, the sections were thoroughly washed with PBST (PBS containing 0.1% Triton X-100), incubated overnight at 4 °C with primary antibodies. After the incubation, the sections were washed with PBST for 15 min and then incubated with the secondary antibody at 37 °C for 1 h. Finally, the nuclei were counterstained with DAPI. Images were acquired at 20 × magnification using both an automatic slice scanning system (Olympus) and a two-photon laser-scanning confocal microscope (Zeiss, Oberkochen, Germany). Image J software was used for analysis.

### Novel-location recognition test

Before the test, mice were acclimated to handling. On day 1, each mouse was placed in the center of a plastic box. In the box, two identical objects (A and B) were positioned at two corners. Each mouse was allowed to explore freely for 5 min. After 24 h, object A remained at the original corner, while object B was placed in a new location. The mouse was re-introduced to the box and given 5 min for exploration. The exploration time for objects A and B, denoted as TA and TB respectively, was recorded. Exploration was defined as the mouse's head being within 3 cm of an object, using a video tracking system (Anymaze Technology SA, Stoelting Co., IL). Mice with TA or TB less than 2 s were excluded from analysis. The discrimination index was calculated as (TB − TA)/(TB + TA). A higher discrimination index indicates better spatial memory retention.

### Morris water maze test

Mice were housed in the test room for 24 h prior to the test. During the training phase, the mice were trained to locate a hidden platform within the Morris water maze. The mice were trained for 5 consecutive days, 3 trials per day (14:00 to 17:00) with a 30-min interval between trials. Each mouse was placed in one of the three quadrants that did not contain the platform, facing the pool wall. If the mouse found the hidden platform within 60 s, it was allowed to stay on the platform for 15 s. If the platform was not found within 60 s, the mouse was gently guided to the platform and allowed to stay there for 15 s. The time taken to find the platform over the 5-day training period was recorded as the escape latency. On day 6, a testing trial was carried out. The hidden platform was removed, and each mouse was placed in the quadrant opposite to the target quadrant. A video tracking system (Chengdu Taimeng Software Co., Ltd, China) was used to record and analyze the time, swimming distance, and trajectory of each mouse in the pool. Mice with any visual or limb impairments were excluded from the analysis.

### Open field test

Mice were handled for 1 day before the test and were placed in the test room the day prior to the behavioral test to acclimatize to the environment. The open field apparatus was a white plastic box measuring 60 × 60 × 50 cm^3^. In the monitoring system, the floor of the box was virtually divided into 16 equal squares, with a central field consisting of the central 4 square regions and 12 peripheral fields. Each mouse was allowed to explore freely in the box for 5 min. The ANY-maze video tracking system (Stoelting Co., WoodDale, IL) was used to record and analyze the time and distance each mouse traveled in different zones.

### Statistical analyses

All data were processed and visualized using GraphPad Prism 8 (La Jolla, CA). Comparisons between two groups were performed using two-tailed unpaired Student’s *t*-tests. Comparisons among multiple groups were performed with one-way, two-way, or repeated measures ANOVA followed by post-hoc tests for multiple comparisons. Statistical significance was set at *P* < 0.05. All values are presented as mean ± SEM.

## Results

### Phosphorylated tau (pTau) accumulation in hippocampal excitatory neurons disrupts the intracellular calcium buffering capacity

**Fig. 1:**
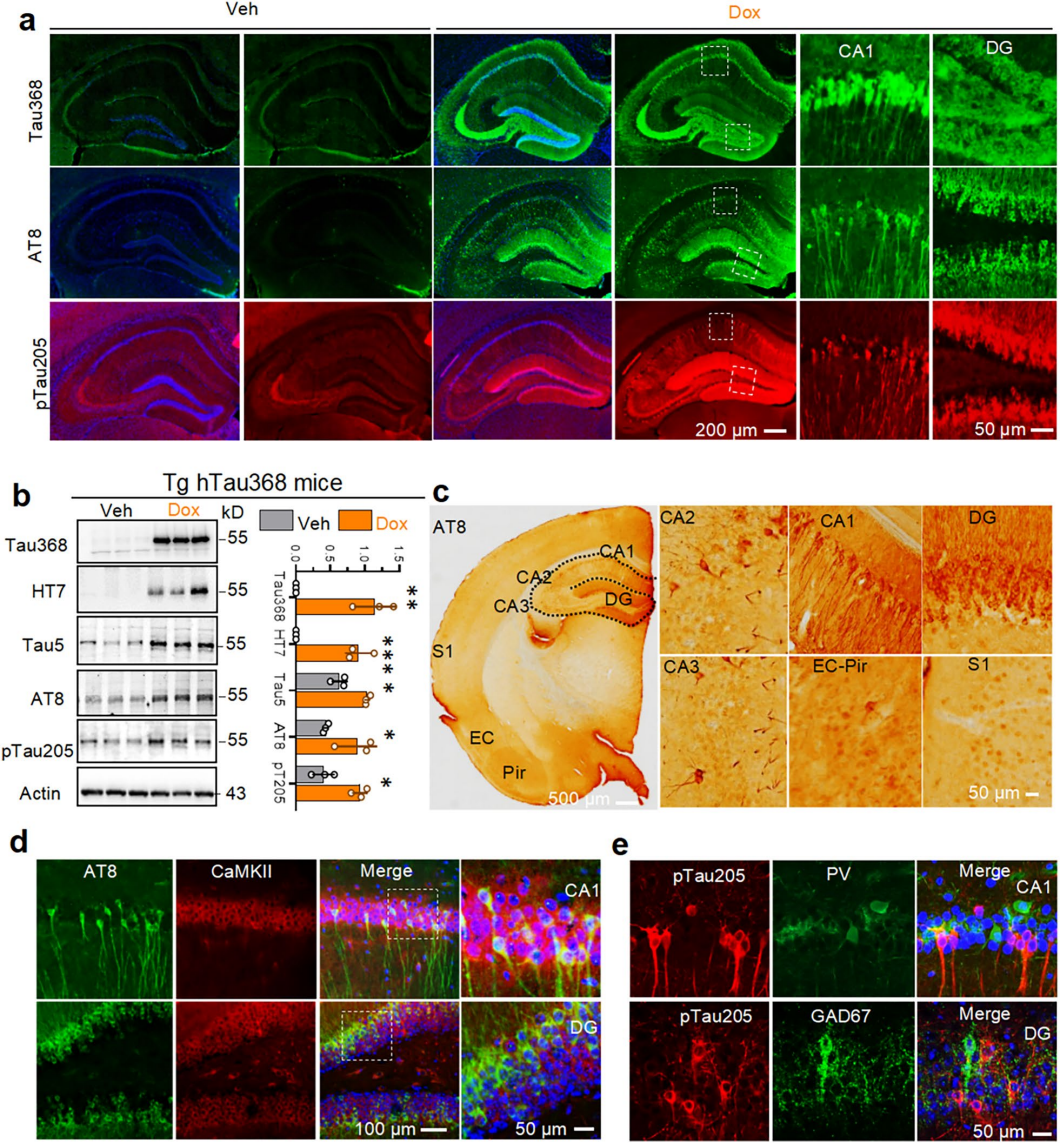
pTau predominately accumulates in hippocampal excitatory neurons of Tg Tau-driven mice. **a-c** Tg hTau368 mice with two-month doxycycline (Dox) treatment exhibited hippocampal-predominant phosphorylated tau (pTau) aggregation (detected by AT8 and pT205-tau antibodies), particularly in the CA1 pyramidal cell layer and DG granule cell layer. (**a**) Representative immunofluorescence (IF) staining. (**b**) Western blotting analysis of RIPA-soluble lysate of hippocampus. Unpaired Student’s* t*-test; *P < 0.05, **P < 0.01, ***P < 0.001. Seven-month-old homozygous Tg hTau368 mice were used (3 mice per group). (**c**) Representative immunohistochemical staining of phosphorylated tau (detected by AT8) aggregation in hippocampal sections from Tg hTau368 mice treated with Dox for 2 months. **d, e** Representative IF staining images of hippocampal sections from hTau368 mice with Dox treatment for 2 months demonstrating that pTau aggregates were localized mainly in CaMKII-positive excitatory neurons, with no colocalization with PV- or GAD67-positive inhibitory neurons. Seven-month-old homozygous Tg hTau368 mice were used.

Truncated tau368 fragments naturally exist in the brain and increase during aging and AD, which are more neurotoxic than other truncated and full-length tau isoforms [33, 34]. We have previously generated the Tg hTau368 mouse model with tetracycline-controlled expression of truncated human tau (hTau368) under the neuronal promoter Eno2 [28]. This Tg mouse model exhibited hippocampal-predominant pTau aggregation (Fig. 1a–c) and cognitive impairments when treated with Dox for 2 months, as previously reported [28]. Immunofluorescence co-staining revealed pTau (AT8 or pTau205) co-localization with CaMKIIα-positive excitatory neurons in hippocampal CA1 and DG regions, with almost no co-localization with parvalbumin (PV)- or GAD67-positive inhibitory neurons (Fig. 1d, e). Consistently, similar tau aggregation patterns were observed in Tg PR5 mice expressing P301L mutant human tau under control of the murine Thy1.2 (Additional file 1: Fig. S1).

**Fig. 2:**
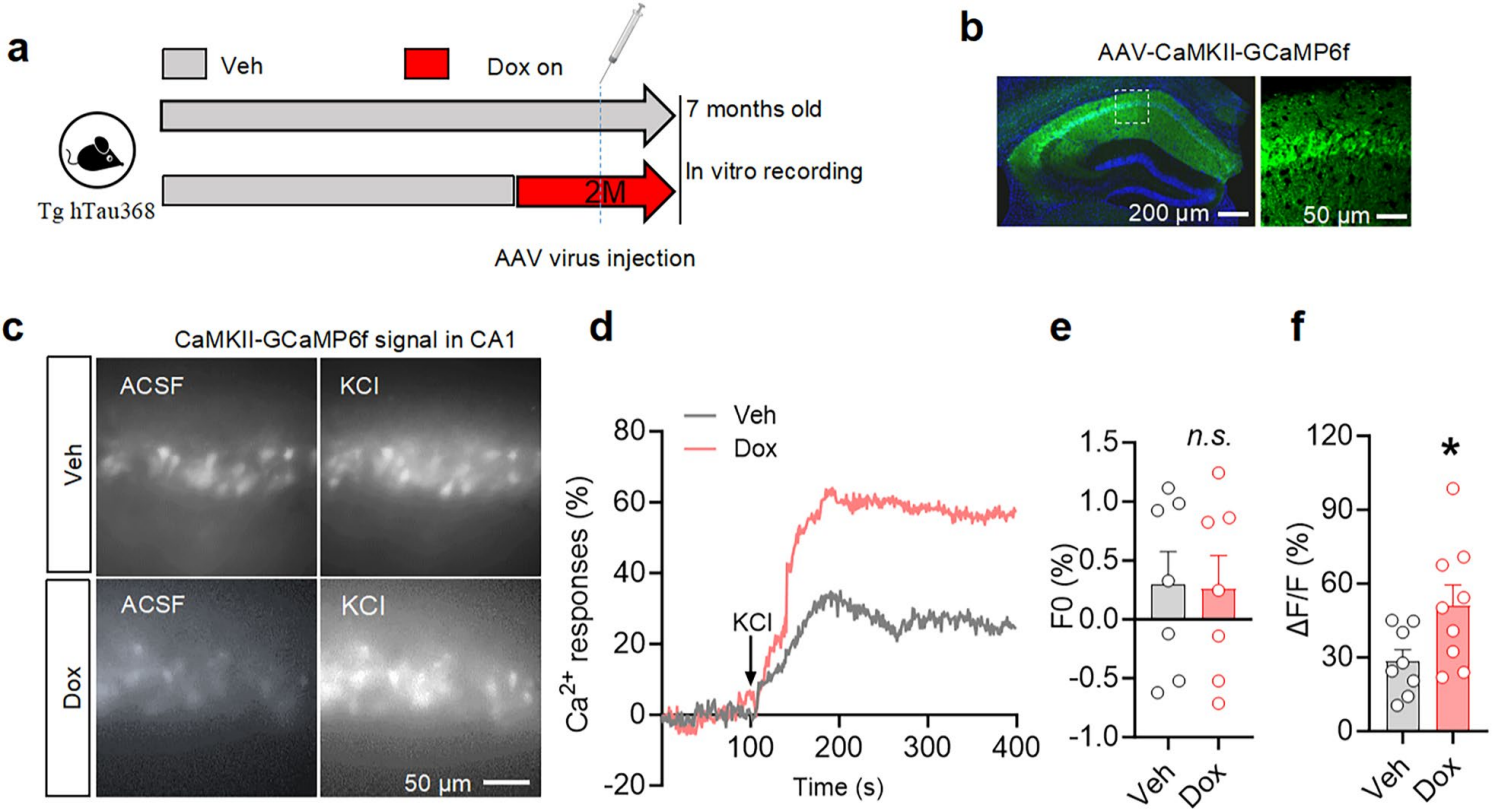
Tau aggregation in hippocampal CA1 excitatory neurons disturbs intracellular calcium signal. **a** Schematic diagram of the experiment. Five-month-old Tg hTau368 mice were treated with Veh/Dox for 2 months. At 1 month before ex vivo calcium signal recording in the hippocampus, the virus AAV-CaMKII-GCamP6f was injected in CA1. **b** Representative IF images of the injection site of AAV-CaMKII-GCaMP6f in CA1. **c, d** Representative images of ex vivo calcium signal recordings of baseline and KCl-induced action potentials in CA1 excitatory neurons in Tg hTau368 mice treated with Veh/Dox for 2 months.** e, f** Quantitative analysis of the baseline fluorescence signals (*F*0) within 100 s before KCl treatment (**e**) and fluorescence signals after KCl treatment (**f**). Unpaired Student’s *t*-test, *n.s.* *P* > 0.05, *P < 0.05. Each point represents a single recording of the mean calcium signal within a field of view. 7-month-old homozygous Tg hTau368 mice, 3–4 mice per group. Data are presented as mean ± SEM

To assess the impact of tau aggregation on calcium dynamics, we injected AAV-CaMKIIα-GCaMP6f into the CA1 of Tg hTau368 mice (Fig. 2a, b). GCaMP6 is a genetically-encoded calcium indicator, composed of cpEGFP (circularly permuted EGFP) protein, CaM (calmodulin), and M13 peptide. The cpGFP protein serves as the source of fluorescence. CaM is sensitive to calcium ions and can bind to them, while the M13 peptide interacts with CaM. The binding of calcium ions to CaM causes a conformational change in CaM that allows its binding to M13. This in turn drives a conformational change in the cpEGFP protein, resulting in a significant enhancement of the fluorescence signal [35]. The KCl-induced depolarization of CA1 neurons (green) resulted in a significantly higher intracellular calcium signal change in the Dox-treated mice (with tau pathology) compared to the Veh group (without tau pathology) (Fig. 2c–f). This suggested that pTau accumulation impaired the calcium buffering capacity of CA1 excitatory neurons. Our findings align with previous research demonstrating elevated cytosolic calcium signals in neurons expressing full-length hTau detected by Fluo-3 AM, another calcium indicator [36, 37].

### Tau aggregation promotes acute seizures induced by hippocampal CA1 and DG hyperexcitability

**Fig. 3:**
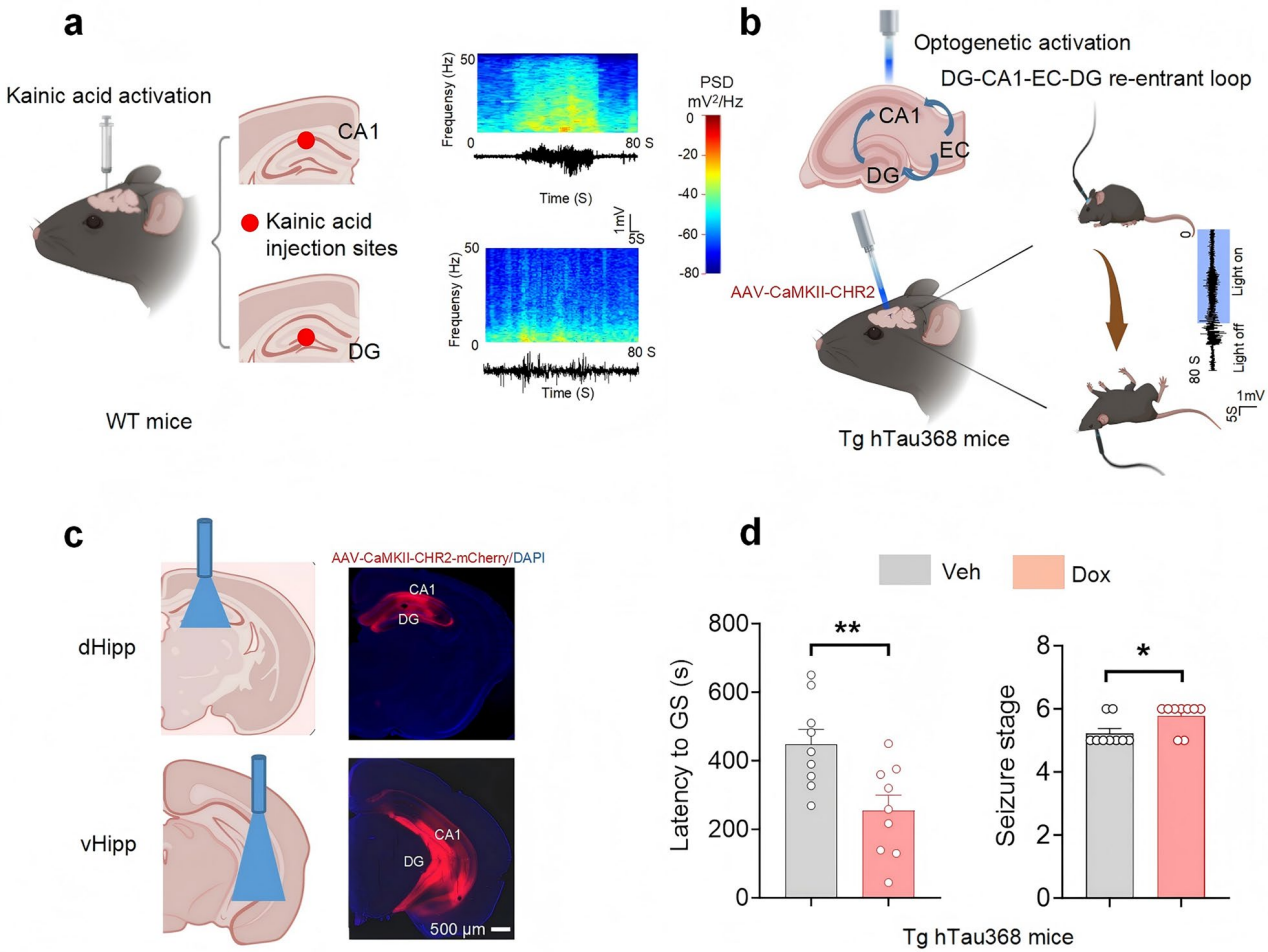
Tau aggregation promotes the susceptibility to acute seizures induced by hyperexcitation of the hippocampal microcircuit. **a** In 16-month-old WT mice, kainic acid (KA, 500 nL, 0.5 μg/μL) was locally injected into CA1 or DG of the hippocampus, and a multi-channel electrode was implanted 0.1 mm above the injection site. Typical epileptic seizure waveforms were successfully recorded in mice. This experiment was repeated three times. **b** Schematic diagram of optogenetic activation of the hippocampal-related epileptic circuit (DG-CA1-EC-DG) to induce epileptic seizures in 16-month-old Tg hTau368 mice. Four weeks after virus injection, blue light was used to over-activate excitatory neurons, which could induce epileptic seizures in aged mice. Typical epileptic seizure waves were recorded in cortex M1. **c** A schematic diagram of the optogenetically activated area (left) and representative images of AAV-CaMKII-ChR2-mCherry injection in the dorsal/ventral hippocampus (right). **d** Aged 16-month-old Tg hTau368 mice treated with Dox for 2 months (from age of 14 months) had a shorter latency to generalized seizures (GS) and a higher seizure stage induced by optogenetic activation. Unpaired Student’s *t*-test, *P < 0.05, *P < 0.01 . Homozygous aged Tg mice were used, 3 mice per group, induction of one seizure per day for 3 consecutive days, totaling 9 seizures. Data are presented as mean ± SEM

To investigate the role of hippocampal CA1 and DG regions in TLE, we injected KA, a natural excitatory neurotoxin derived from red algae and an agonist of ionotropic glutamate receptors (AMPA and KA receptors), into the CA1 or DG of aged wild-type mice. KA injection in both regions induced epileptic seizures and typical epileptic seizure waveforms (Fig. 3a and Additional file 1: Fig. S2), mimicking human TLE [38]. This suggests that the KA-mediated hyperactivation of CA1 and DG neurons contributes to acute seizures.

To further elucidate the role of excitatory neurons in CA1 and DG and the influence of tau pathology on acute seizures, we injected AAV-CaMKIIα-ChR2-mCherry into the CA1 and DG regions of aged Tg hTau368 mice. Four weeks post-injection, blue light stimulation was used to activate neurons in these regions. Both dorsal (dHipp) and ventral (vHipp) hippocampal stimulation induced seizures of varying severity and in vivo electrophysiological recording revealed typical epileptic seizure waveforms (Fig. 3b, c, Additional file 2: Supplementary Video 1). Seizures ceased upon cessation of light stimulation, and the mice returned to normal behavior gradually (Additional file 2: Supplementary Video 1). The Dox group exhibited shorter seizure latency (Fig. 3d, Additional file 1: Fig. S3) and higher seizure severity compared to the Veh group (Fig. 3d), indicating that tau pathology enhances the susceptibility to acute seizures. The hippocampal-parahippocampal circuits, particularly the DG-CA1-EC-DG loop, play a critical role in TLE initiation and propagation [18]. Our findings suggest that abnormal activation of CA1 and DG excitatory neurons drives acute seizures, and that tau aggregation increases TLE susceptibility, potentially through hyperexcitability of the DG-CA1-EC-DG circuit.

### Tau pathology correlates with cerebral hypermetabolism, hyperexcitable behavioral phenotypes and cognitive deficits

**Fig. 4:**
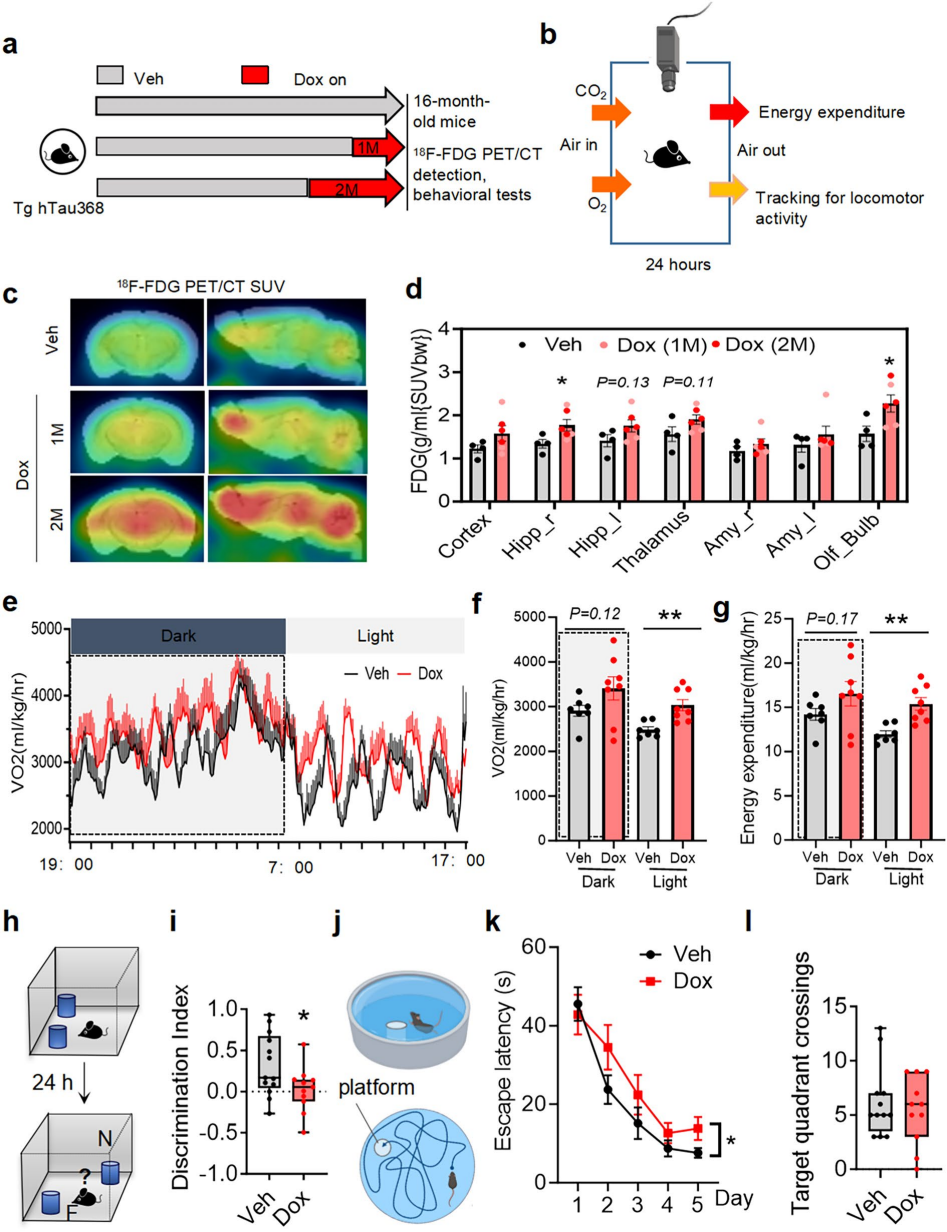
The aged Tg Tau-driven mice show brain hypermetabolism, higher energy expenditure and cognitive deficits. **a** Schematic diagram of the experiment. **b** Schematic diagram of mouse energy metabolism and activity monitoring for a whole day. **c, d** Representative images of brain ^18^F-FDG PET/CT examination (**c**) and average ^18^F-FDG uptake (**d**) in multiple brain regions of 16-month-old homozygous hTau368 transgenic mice. Unpaired Student’s *t*-test, *P < 0.05, 3–4 mice per group. Data are presented as mean ± SEM. **e–g** Oxygen consumption (**e**) every 5 min at different times within 24 h and quantitative statistics of (**f**) mean oxygen consumption and (**g**) energy expenditure of aged Tg hTau368 mice treated with Veh/Dox for 2 months, during night (Dark, 19:00–7:00) and day (Light, 7:00–19:00). Unpaired Student’s *t*-test, **P < 0.01, 7–8 mice per group. Data are presented as mean ± SEM. **h** Schematic illustration of the the novel-location recognition test. F stands for familiar; N stands for novel. **i** Aged Tg hTau368 mice with Dox treatment for 2 months showed poorer performance in discriminating the object removed to a new place in the novel-location recognition test. **j** Schematic illustration of Morris-water maze. **k, l** Aged Tg hTau368 mice with Dox treatment for 2 months showed a longer latency to find the platform during training (days 1–5) (**k**) and comparable target quadrant crossings after training (day 6) (**l**). Repeated measures ANOVA followed by Tukey’s *post-hoc* test for **k**. Unpaired Student’s *t*-test for **i** and **l**. *P < 0.05. 16-month-old homozygous aged hTau368 mice, 12 mice per group. Data are presented as mean ± SEM

Increases of neuronal and brain network excitability are age-dependent. Dysregulation of homeostatic control of neural microcircuits in aged mice is associated with cognitive impairment [39]. To investigate the effects of tau pathology on aging as AD is an age-dependent disease, we induced hTau expression in 14–15-month-old Tg hTau368 mice by administering 1–2 months of Dox treatment, and then ^18^F-FDG PET/CT scanning, energy metabolism, activity monitoring and cognitive tests were conducted (Fig. 4a, b). ^18^F-FDGPET/CT imaging reflects the intensity of glucose metabolism in the brain, and to a certain extent, the excitability state of the whole brain, which has a certain diagnostic value for AD progression [40, 41]. ^18^F-FDG PET/CT scanning in aged Tg hTau368 mice (16 months) revealed elevated glucose metabolism in the hippocampus and olfactory bulb following 1–2 months of Dox treatment (Fig. 4c, d). This is consistent with the early-stage hypermetabolic states observed in young 3 × Tg (7 months old, carrying the APP/PS1/P301L tau mutation) and PR5 (3 months old, carrying the tau P301L mutation) mice [42–44].

The cerebral hypermetabolism paralleled increased O_2_ consumption (Fig. 4e, f), energy expenditure (Fig. 4g), and spontaneous movements (Additional file 1: Fig. S4), especially during the daytime at resting state of mice. Cognitive deficits were also found in Dox-treated Tg hTau368 mice, manifested as a lower novel location discrimination index in the novel-location recognition test (Fig. 4h, i) and a longer latency to find the platform in the Morris water maze test (Fig. 4j-l). Additionally, the aged Tg hTau368 mice with 2 months of Dox treatment exhibited tauopathology, neuronal loss and glial activation in the hippocampus (Additional file 1: Fig. S5). The results above suggest that tau pathology is associated with elevated cerebral metabolism, hyperexcitable behavioral phenotype and cognitive deficits in aged mice.

### Tau pathology induces CB reduction and synaptic dysfunction

**Fig. 5:**
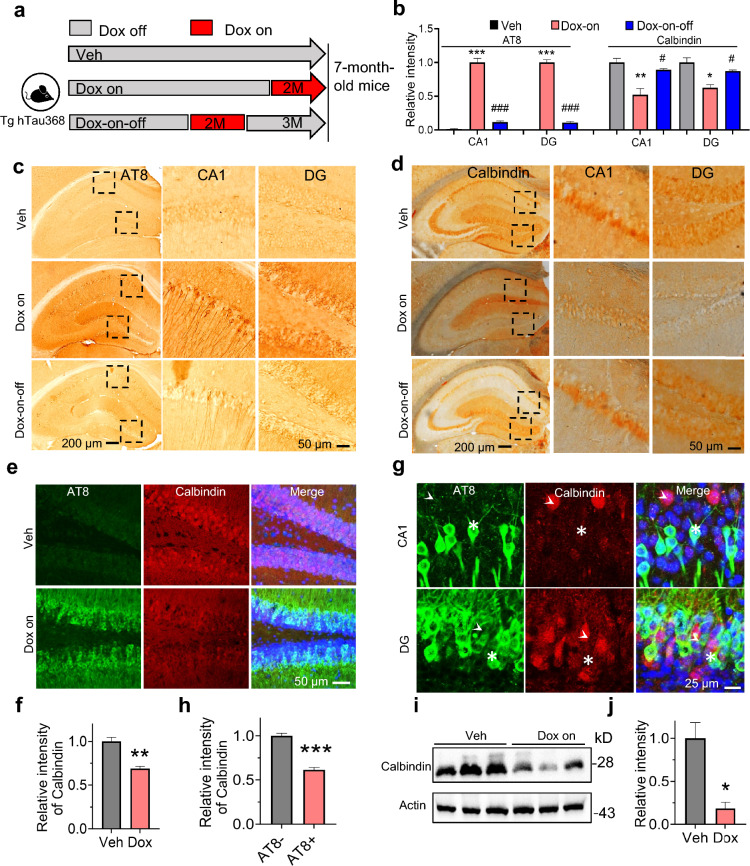
Tauopathy downregulates CB over expression in the hippocampus also amelioratived the proliferation of Iba1+ microglia caused by tauopathy. **a** Schematic diagram of the experiment. **b–d** In Tg hTau368 mice treated with Dox for 2 months (Dox-on), tau aggregation occurred in the hippocampal CA1 and DG regions, and the levels of calbindin-D28k (CB) in hippocampal CA1 and DG were reduced. However, after Dox withdrawal for 3 months (Dox-on–off), the tau pathology was cleared, accompanied by recovery of CB expression. Representative images of IHC staining of AT8 (**c**) and calbindin-D28k (**d**), and quantitative analysis (**b**). One-way analysis of variance, followed by post hoc Tukey’s multiple comparison test. *P < 0.05, **P < 0.01, ***P < 0.001 *vs* Veh group, ^*#*^*P* < 0.05*, *^*##*^*P* < 0.01*, *^*###*^*P* < 0.001 *vs* Dox-on group. **e, f** IF co-staining of AT8 and CB in the DG region (**e**), and quantitative analysis (**f**). Unpaired Student’s *t*-test, **P < 0.01. *n* = 3 mice per group. **g, h** Representative magnified images of IF co-staining of AT8 and CB in the CA1 and DG regions of the Dox-on group (**g**), and (**h**) quantitative analysis. Asterisks represent AT8-positive neurons, and arrowheads represent AT8-negative neurons. Unpaired Student’s *t*-test, ***P < 0.001, 3 mice per group. **i, j** Western blot of RIPA-soluble lysate of hippocampal tissue in the Veh group and the Dox-on group, and quantitative statistics. Unpaired Student’s *t*-test, *P < 0.05, 3 mice per group. Seven-month-old homozygous Tg hTau368 mice were used. Data are presented as mean ± SEM

To explore the effect of tau pathology on CB and synapse-associated proteins, hTau expression was controlled via the Tet-on system in Tg hTau368 mice, i.e., hTau was expressed when Dox was given (Dox-on), and when Dox was withdrawn, hTau expression ceased and was gradually cleared (Dox-on–off). The phenomenon has also been reported in other Tg lines [45, 46]. As expected, tau pathology in the Dox-on–off mice was largely cleared after 3 months, especially in CA1 pyramidal neurons and DG granule cells (Fig. 5a–c). We also observed a rebound in CB expression (Fig. 5d) and increased expression of synaptic proteins (Additional file 1: Fig. S6), such as postsynaptic density protein 95 (PSD95) and synapsin 1 (SYN-1). Immunofluorescent staining and Western blotting data also confirmed CB reductions in the Dox-on group compared with Veh group (Fig. 5e, f, i and j), as well as in the AT8-positive neurons compared with in the AT8-negative neurons (Fig. 5g, h).

### Upregulation of CB alleviates the tau pathology-induced neuronal hyperexcitability, neuroinflammation and cognitive impairment

**Fig. 6:**
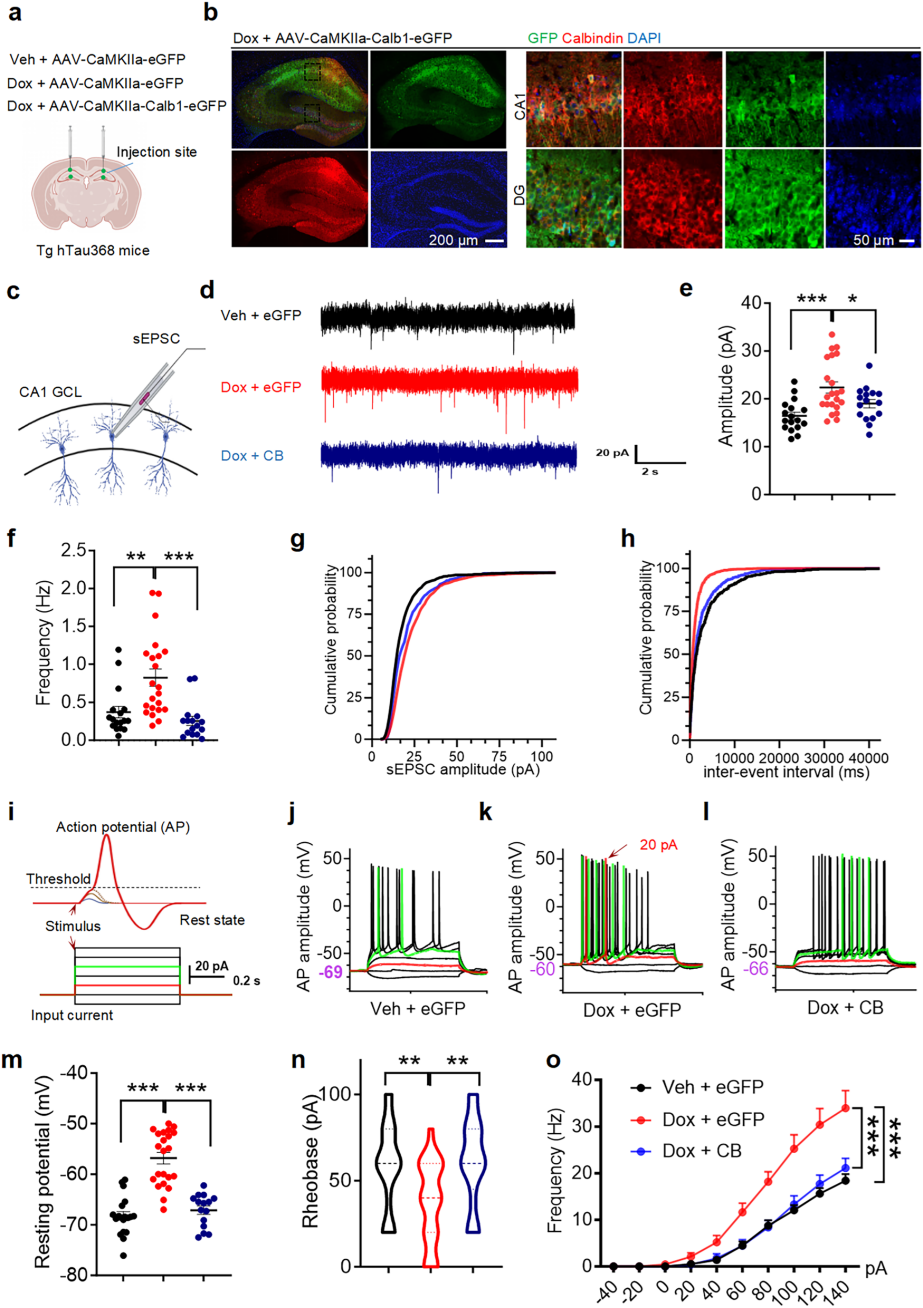
Overexpression of CB in hippocampal excitatory neurons alleviates neuronal hyperexcitation caused by tau aggregation. **a** Schematic diagram of virus injection in CA1and DG of three groups. **b** Overview images of hippocampal CB immunostaining and virus expression (left), and magnified images in CA1 and DG (right) in the Dox + CB group. **c, d** Schematic diagram of patch-clamp recording of spontaneous excitatory postsynaptic currents (sEPSCs) in excitatory neurons of the CA1 pyramidal cell layer (**c**), and representative recordings of the three groups (**d**). **e–h** Quantitative statistics of the amplitude (**e**) and firing frequency (**f**) of sEPSCs in the three groups, and the corresponding cumulative probability curves (**g, h**). **i–l** Schematic diagram of patch-clamp recording of evoked action potentials in the three groups (**i**), and representative images in the three groups (**j–l**). An input current of 20 pA (marked in red) induced action potentials in the Dox + eGFP group (**k**), but not in the Veh + eGFP (**j**) and Dox + CB group (**l**). **m–o** Quantitative statistics of the resting membrane potential (**m**), rheobase (**n**), and action potential firing frequency (**o**). One-way analysis of variance, followed by post-hoc Tukey’s multiple comparison test. *P < 0.05, **P < 0.01, ***P < 0.001. Data are presented as mean ± SEM. Each point represents a single recording of one neuron. Seven-month-old homozygous Tg hTau368 mice, 4–5 mice per group

To investigate whether CB defects mediate tau-associated neuronal hyperexcitability, we made electrophysiological recordings in the CA1 CaMKII-eGFP-positive pyramidal neurons of Tg hTau368 mice treated with Veh + AAV-CaMKIIa-eGFP (Veh + eGFP), Dox + AAV-CaMKIIa-eGFP (Dox + eGFP), and Dox + AAV-CaMKIIa-Calb1-eGFP (Dox + CB) (Fig. 6a and Additional file 1: Table S1). In these groups, 5-month-old homozygous Tg hTau368 mice received Veh or Dox treatment for 2 months. One and a half months prior to patch-clamp recording, the virus AAV-CaMKIIa-eGFP, AAV-CaMKIIa-eGFP, or AAV-CaMKIIa-Calb1-eGFP was injected into the hippocampal CA1 and DG. CB expression was confirmed in CA1 and DG neurons in the Dox + CB group (Fig. 6b). Tau accumulation (Dox + eGFP group) increased sEPSC amplitude and frequency, indicating elevated neuronal excitability, while CB overexpression reversed these effects (Fig. 6c–h). Additionally, neurons in the Dox + eGFP group exhibited higher resting membrane potentials, lower rheobase, and increased frequency of evoked action potentials, all of which were normalized by CB overexpression (Fig. 6i–o). CB overexpression in the hippocampus also ameliorated the proliferation of Iba1^+^ microglia caused by tauopathy (Additional file 1: Fig. S7). These findings suggest that CB overexpression alleviates the tau accumulation-induced hyperexcitability and neuroinflammation.

**Fig. 7:**
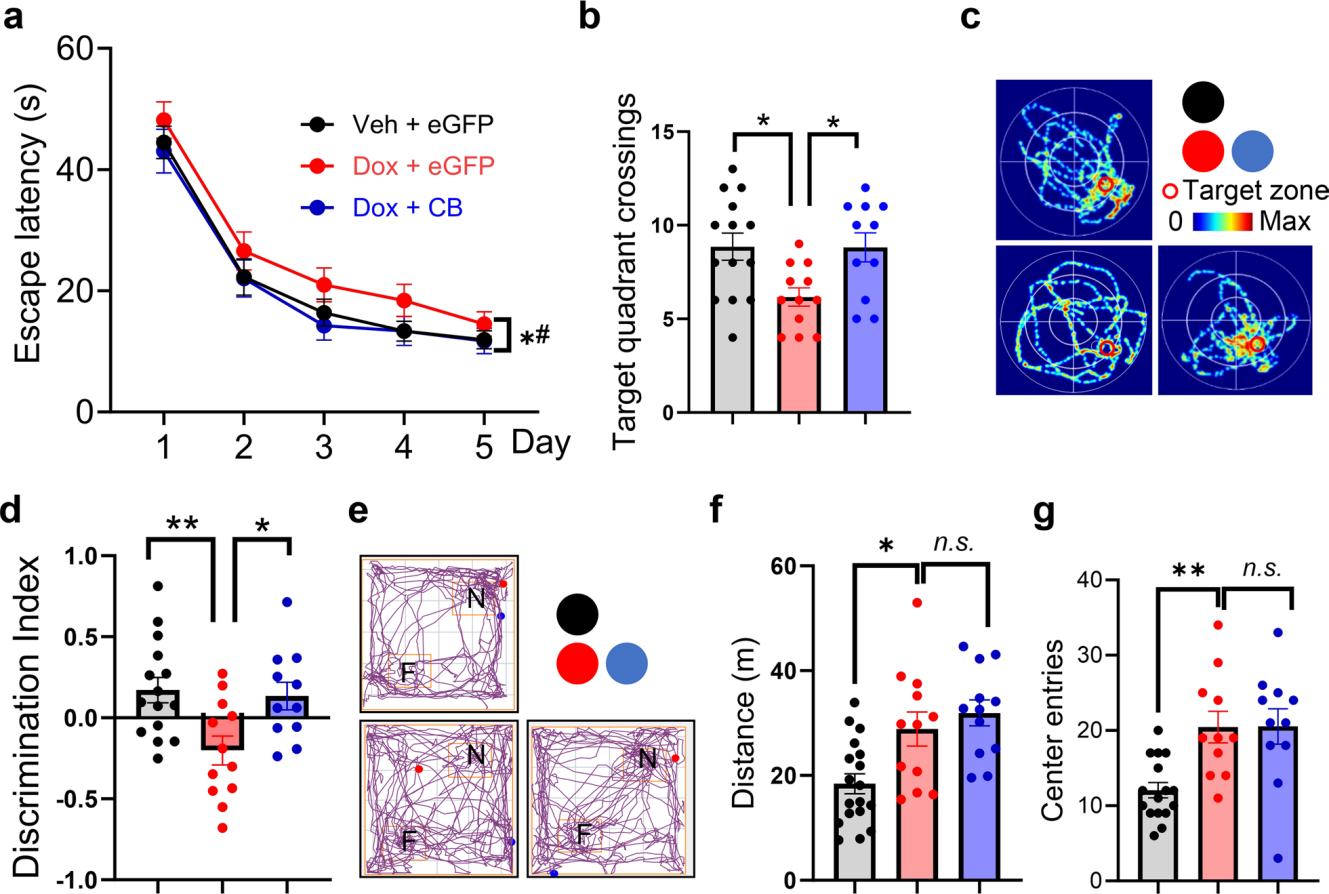
Upregulation of CB alleviates cognitive deficits caused by hippocampal tau aggregation. **a–c** The latency to find the platform during training on days 1–5 (**a**), the number of target quadrant crossings on day 6 (**b**), and representative images of swimming tracks (**c**) in the Morris water maze test. *P < 0.05, Veh + eGFP vs Dox + eGFP group, ^*#*^*P* < 0.05, Veh + eGFP vs Dox + CB group. **d, e** Discrimination ability and movement tracks in the novel-location recognition test. **f, g** The total movement distance (**f**) and the number of times crossing the central area (**g**) in the open field test. Two-way repeated-measures analysis of variance was used for **a**. One-way analysis of variance followed by the post-hoc Tukey’s multiple comparison test. *P < 0.05, **P < 0.01*.* Data are presented as mean ± SEM. F, familiar; N, novel. The experiments were conducted in 7-month-old homozygous Tg hTau368 mice, with the following group sizes: Veh + eGFP (*n* = 15), Dox + eGFP (*n* = 12), and Dox + CB (*n* = 12)

Behavioral tests also revealed improvement of cognitive performance of Dox-treated Tg hTau368 mice with CB overexpression. In the Morris water maze, the Dox + CB group showed a tendency of reduced latency to find the platform (Fig. 7a) and significantly increased target quadrant crossings compared to the Dox + eGFP group (Fig. 7b, c). Similarly, in the novel-location recognition test, the Dox + CB group demonstrated better recognition of the novel location (Fig. 7d, e). However, CB overexpression did not affect spontaneous behaviors in the open field test (Fig. 7f, g). Overall, CB overexpression in hippocampal CA1 and DG excitatory neurons mitigates the tau-induced cognitive dysfunction, highlighting its therapeutic potential.

### Reduced CB expression correlates with cognitive deterioration and an advanced disease stage in AD patients

**Fig. 8:**
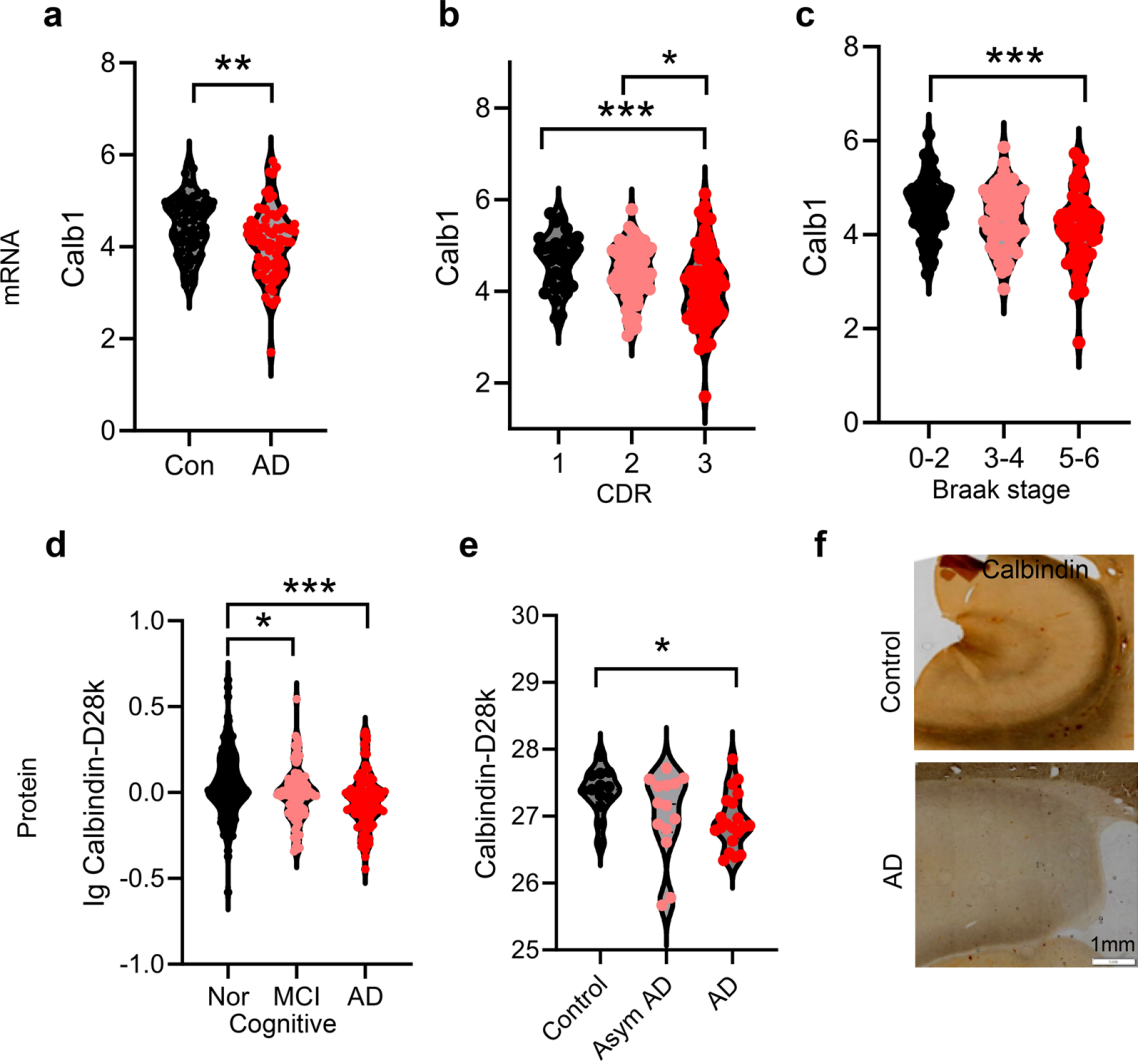
CB deficiency is correlated with cognitive deterioration in AD patients. **a–e** mRNA expression of *CALB1* was decreased in the brain tissues of AD patients (**a**), and correlated with the Clinical Dementia Rating Scale score (**b**) and severity of the clinical Braak staging (**c**). The decrease of CB expression in the brain tissues of AD patients was related to cognitive decline (**d**) and the severity of clinical symptoms (**e**). One-way analysis of variance followed by post-hoc Tukey’s multiple comparison test. *P < 0.05, **P < 0.01, ***P < 0.001. Data are presented as mean ± SEM. Raw data were from http://www.alzcode.xyz/. **f** Immunohistochemical staining revealed a marked reduction of CB expression in hippocampal tissue from a 65-year-old AD patient compared with an age-matched individual without AD. Con: normal control; Nor: normal cognitive function; MCI: mild cognitive impairment; Asym AD: AD patients in the early pre-clinical stage; AD: AD patients in the dementia stage; CDR: Clinical Dementia Rating Scale

To investigate the association between CB expression and cognitive decline in AD patients, we analyzed CB expression using a publicly available AD database (http://www.alzcode.xyz/) [47]. Compared to healthy controls, AD patients exhibited significantly decreased CB transcript levels in the brain (Fig. 8a). To evaluate disease progression, patients were stratified by cognitive function (Clinical Dementia Rating [CDR] scale; Fig. 8b) and neuropathological severity (Braak staging; Fig. 8c). Strikingly, CB transcript levels progressively declined with worsening cognitive impairment (higher CDR scores) and advanced Braak stages. Consistent with transcriptional changes, the total CB protein level was also markedly reduced in AD patients (Fig. 8d, e). Immunohistochemical staining confirmed diminished CB levels in the hippocampal region of AD brains (Fig. 8f). These findings collectively highlight a robust inverse correlation of CB depletion with both cognitive decline and AD progression. Further studies should focus on specific cell types by single-cell transcriptomics and proteomics sequencing.

## Discussion

**Fig. 9:**
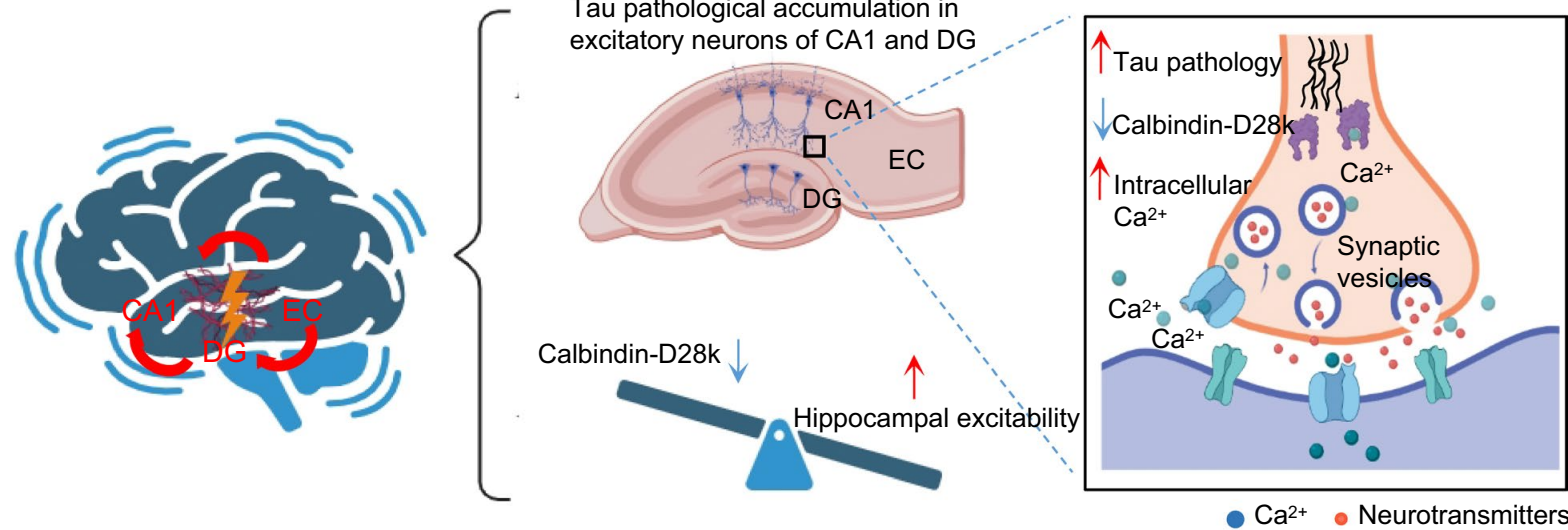
An illustration showing that tau aggregation in hippocampal CA1 and DG excitatory neurons reduces CB expression, leading to increased epileptic susceptibility and cognitive impairment in the tau-driven mouse model. Figure was created by BioRender.com

Emerging evidence suggests that the hippocampal network hyperexcitability caused by excitation-inhibition imbalance occurs in early AD progression and exacerbates cognitive decline, though the underlying mechanisms remain elusive. Our study demonstrated that tau aggregation within hippocampal CA1 and DG excitatory neurons leads to reduced CB expression, elevated intracellular calcium transients, and enhanced neuronal network excitability, ultimately increasing the susceptibility to seizures and cognitive impairment. Notably, CB overexpression in hippocampal CA1 and DG excitatory neurons ameliorates the tauopathy-induced hyperexcitability and cognitive deficits in mice (Fig. 9). Clinical correlation analysis from AD databases further revealed association of CB deficiency with accelerated cognitive decline and increased disease severity. These findings elucidate that CB deficiency mediates the tau-related epileptogenesis and cognitive dysfunction at both circuit and molecular levels, providing a novel therapeutic target for AD intervention.

Tau is a microtubule-associated protein that plays a pivotal role in maintaining microtubule assembly and stability. Under physiological conditions, tau exists predominantly in an unstructured conformation with low intrinsic aggregation propensity. However, pathological hyperphosphorylation drives its misfolding into paired helical filaments and NFTs, which further form large aggregates—a defining feature of tauopathies. These pathologies are not exclusive to AD but are also implicated in a spectrum of neurodegenerative disorders, including progressive supranuclear palsy, corticobasal degeneration, argyrophilic grain disease, Pick’s disease, Huntington’s disease, and frontotemporal dementia with Parkinsonism linked to chromosome 17 [48, 49]. Notably, tau pathology has been identified in the hippocampus of TLE patients [4, 50], suggesting a shared mechanism between AD and TLE. Both disorders exhibit hippocampal network hyperexcitability during early disease stages [4].

To investigate the role of hippocampal microcircuits in acute seizure, we used two methods to induce acute seizures: injection of KA to activate CA1 and DG subregions, and selective optogenetic stimulation of excitatory neurons. Our results revealed that overactivation of CA1/DG neurons reliably induced seizures in mice, underscoring the critical contribution of hippocampal hyperexcitability to acute seizure susceptibility. In the optogenetic model, CaMKIIα promoter-driven expression of channelrhodopsin-2 (ChR2) enabled precise spatiotemporal control of neuronal activity. Blue light (475 nm) triggered rapid cation influx (e.g., Na⁺) through ChR2, eliciting action potentials and transient hippocampal overexcitation. Termination of photostimulation promptly closed the channels, allowing neuronal activity to return to baseline [31]. The Dox-treated Tg hTau368 mice exhibited significantly reduced seizure thresholds and more severe phenotypes upon optogenetic induction, further supporting tau pathology as a driver of acute seizure vulnerability. This phenomenon recapitulated those in other Tg mouse models expressing mutant tau variants such as PS19 (P301S), Tau22 (G272V/P301S), and rTg4510 (P301L) [51, 52]. Complementary studies in *Mapt*⁻/⁻ mice demonstrated that tau ablation reduces action potential firing, lowers the cortical excitation-inhibition (E/I) ratio, and enhances excitability of inhibitory neurons, collectively mitigating neuronal hypersynchronization and seizure incidence [53]. These findings highlight tau-driven E/I imbalance as a central mechanism underlying the circuit-level hyperexcitability in epilepsy.

Conversely, some studies indicate that tau pathology may also drive hypoexcitability [54], and suppress Aβ-driven cortical hyperactivation [55]. Phosphorylation of different tau residues has different effects on firing frequency of primary hippocampal neurons [54]. The role of tau in mediating neuronal excitability changes may vary depending on tau features (mutation, phosphorylation, isoform), the brain region and the disease stage [10]. In the present study, we showed that truncated hTau368 enhances the activity of CA1 excitatory neurons during the early stage of tau pathology. This neuronal hyperexcitability is accompanied by a hypermetabolic state in the hippocampus, as detected by ^18^F-FDG PET/CT, together with systemic hypermetabolism and increased spontaneous locomotor activity. Head-to-head comparison in diverse tauopathy models (e.g., P301L, P301S) is needed to further validate the current hypotheses.

Under resting conditions, extracellular calcium concentrations (1.1–1.4 mM) starkly contrast with intracellular levels (50–300 nM), yet neuronal activation rapidly elevates cytosolic calcium to micromolar ranges [56]. Calcium homeostasis is tightly regulated by its influx through plasma membrane channels, buffering by calcium-binding proteins such as CB, and buffering by endoplasmic reticulum (ER) [57]. Electrophysiological recordings and GCaMP6f-mediated calcium imaging provided a robust method to study the interplay between tau, calcium, and neuronal activity. Our data demonstrated that tau pathology exacerbated intracellular calcium transients and CA1 neuronal hyperexcitability, aligning with prior in vitro observations. Full-length tau overexpression similarly disrupts calcium handling and induces ER stress [37]. *PS1* and *PS2* double knockout or expression of an FAD mutation in mouse hippocampal neurons increased ER Ca^2+^ levels and consequently elevated Ca^2+^ release into the cytoplasm in the presence of a stimulus [58]. Notably, CB overexpression in CA1 neurons rescued the tau-induced hyperexcitability, suggesting CB deficiency as a key mediator.

CB exhibits high calcium affinity and serves as a critical buffer against cytosolic calcium overload. CB also modulates action potential kinetics and synaptic transmission [24, 59]. CB is enriched in CA1 pyramidal neurons and DG granule cells, with sparse expression in CA1 interneurons [26, 60, 61]. Anatomically, CB is localized within axons and dendritic spines of neurons, where it exerts a dynamic regulatory effect on synaptic plasticity. At the presynaptic terminals, CB plays a promoting role in vesicle release and paired-pulse facilitation [62]. Conversely, in the postsynaptic context, CB is indispensable for the induction and maintenance of long-term potentiation in CA1 and DG excitatory neurons [63]. By binding excess calcium ions, CB effectively down-regulates the intracellular free calcium levels, thereby safeguarding neurons from the potentially lethal effects of calcium overload [28]. Moreover, the regulatory function of CB extends to influencing the firing of neuronal action potentials and the intricate processes of synaptic neurotransmitter transport and release. In our study, tauopathology-associated calcium surges in CA1 neurons are correlated with diminished CB expression, likely due to impairment of buffering capacity. The pTau aggregates are localized exclusively to CA1/DG excitatory neurons and coincide with CB downregulation, leading to calcium dysregulation, mitochondrial stress, oxidative damage, and apoptotic signaling [37, 59]. Disruption of calcium homeostasis further alters neurotransmitter release and transcriptional programs, exacerbating the E/I imbalance [22]. Remarkably, after Dox withdrawal for three months, tau pathology was relieved, and CB levels and synaptic transmission were restored, accompanied by cessation of hippocampal neurons loss [28] and cognition recovery [28]. Similarly, AAV-mediated CB overexpression in Tg hTau368 mice attenuated tauopathology-induced cognitive deficits, implicating CB restoration as a viable therapeutic strategy. Our data established connections among tauopathy, CB deficiency, calcium homeostatic imbalance, synaptic dysfunction and cognitive deficits.

Some limitations need to be noted in this study. First, the molecular link between tau pathology and CB deficiency remains unclear. A previous study indicated that the transcription factor ΔFosB is induced in the hippocampus of AD/TLE and mediates epigenetic silencing of CB [27]. In addition, tau accumulation activates the JAK2–STAT1 pathway [64], but whether this pathway is linked to *Calb1* transcriptional repression warrants further investigation. Alternative pathways that are involved in the effect of tau accumulation on calcium homeostasis should also be addressed, including ion channel dysregulation, ER-mitochondrial Ca^2^⁺ transfer overload, and altered levels of other calcium-binding proteins. Ye et al. found that intracellular tau could upregulate mRNA and protein levels of TRPC1 (transient receptor potential channel 1), with an activated store-operated calcium entry, and an increased intraneuronal steady-state [Ca^2+^]_i_ [37]. Yin et al. reported that hTau accumulation causes calcium-dependent, protein phosphatase calcineurin-mediated dephosphorylation/inactivation of CaMKIV (calcium/calmodulin-dependent protein kinase IV)–CREB (cAMP response element binding protein) signaling in the nuclei, resulting in synaptic and memory deficits [65]. In vivo two-photon calcium imaging during spontaneous activity will be necessary to directly record calcium dysregulation in the intact hippocampal circuit. Second, the role of CB in interneurons remains unexplored, despite evidence that interneuron-specific CB knockdown protects against memory deficits [25]. Distinct inhibitory neuron subtypes (SST^+^ and LAMP5^+^) are enriched in AD patients with preserved cognitive function in late life, whereas somatostatin-expressing inhibitory neurons are selectively depleted during disease progression [66]. As interneurons contribute to network remodeling in the hippocampus [67], the selective vulnerability of interneurons to tauopathy leading to local excitatory-inhibitory imbalance should also be addressed. Finally, whether there is an interaction between Aβ and tauopathy, along with its influence on the hippocampal neural circuit, still requires further exploration.

## Conclusion

In conclusion, our study demonstrated that tau selectively accumulates in hippocampal excitatory neurons, resulting in increased neuronal excitability and a heightened susceptibility to TLE. The concomitant reduction in CB expression within these neurons mediates the hyperexcitability and the cognitive dysfunction associated with tau pathology. These findings underscore the potential of targeting CB-mediated calcium homeostasis as a therapeutic strategy for AD.

## Supplementary Information

**Additional file 1**: **Fig. S1**. pTau accumulation in hippocampal excitatory neurons of PR5 mice. **Fig. S2**. In vivo electrophysiological recording after KA local injection. **Fig. S3**. Aged 16-month-old Tg hTau368 mice treated with Dox for 2 months had a shorter latency to generalized seizures induced by optogenetics. **Fig. S4**. Increased locomotor activity in aged Tg hTau368 mice. **Fig. S5**. Tau accumulation, neuronal loss and glial activation in the hippocampus of aged Tg hTau368 mice with 2 months of Dox treatment. **Fig. S6**. Hippocampal tau aggregation correlated with reduced CB and synapse-related proteins. **Fig. S7**. Overexpression of CB in the hippocampus ameliorates neuroinflammation caused by tauopathology. **Table S1**. Viruses and injection sites in this study. **Screenshot of Video 1**.

**Additional file 2**: **Supplementary Video 1**. Optogenetic induction of seizures and in vivo electrophysiological recording.

## Data Availability

All data provided in this paper are available from the leading contact, Prof Jian-Zhi Wang upon reasonable request.

## Abbreviations

AD: Alzheimer’s disease
TLE: Temporal lobe epilepsy
CB: Calbindin-D28k
PET/CT: Positron emission tomography/Computed tomography
AAV: Adeno-associated virus
CA1: Cornu Ammonis 1
DG: Dentate gyrus
CaMKII: Calcium/calmodulin-dependent protein kinase II
pTau: Tau phosphorylation
NFTs: Neurofibrillary tangles
EC: Entorhinal cortex
KA: Kainic acid
GS: Generalized seizures
RQ: Respiratory quotient
AP: Action potential
sEPSCs: Spontaneous excitatory postsynaptic currents
CDR: Clinical Dementia Rating
ER: Endoplasmic reticulum
E/I: Excitatory-inhibitory
VO₂: Oxygen consumption
VCO_2_: Carbon dioxide production
