# Supplementary material for: Calbindin-D28k deficiency mediates tau-driven hippocampal hyperexcitement and cognitive impairment
Source: Transl Neurodegener. 2026 May 12;15:22. doi: 10.1186/s40035-026-00547-3 (PMC13162517; doi:10.1186/s40035-026-00547-3)
Supplement: Supplementary file 1 — Additional file 1: Fig. S1. pTau accumulation in hippocampal excitatory neurons of PR5 mice. Fig. S2. In vivo electrophysiological recording after KA local injection. Fig. S3. Aged 16-month-old Tg hTau368 mice treated with Dox for 2 months had a shorter latency to generalized seizures induced by optogenetics. Fig. S4. Increased locomotor activity in aged Tg hTau368 mice. Fig. S5. Tau accumulation, neuronal loss and glial activation in the hippocampus of aged Tg hTau368 mice with 2 months of Dox treatment. Fig. S6. Hippocampal tau aggregation correlated with reduced CB and synapse-related proteins. Fig. S7. Overexpression of CB in the hippocampus ameliorates neuroinflammation caused by tauopathology. Table S1. Viruses and injection sites in this study. Screenshot of Video 1. [file 40035_2026_547_MOESM1_ESM.docx]

**Supplementary materials**


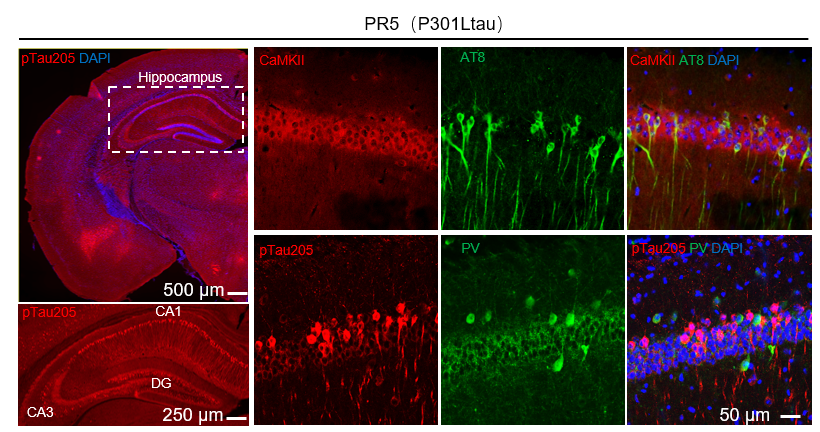


**Figure S1. pTau accumulation in hippocampal excitatory neurons of PR5 (P301Ltau) mice.** Seven-month-old PR5 mice exhibited hippocampal-predominant phosphorylated tau (pTau) aggregation (detected by pT205-tau antibodies), particularly in the CA1 pyramidal cell layer and DG granule cell layer. pTau aggregates localized mainly to CaMKII-positive excitatory neurons, almost no colocalization was observed in PV- positive inhibitory neurons.


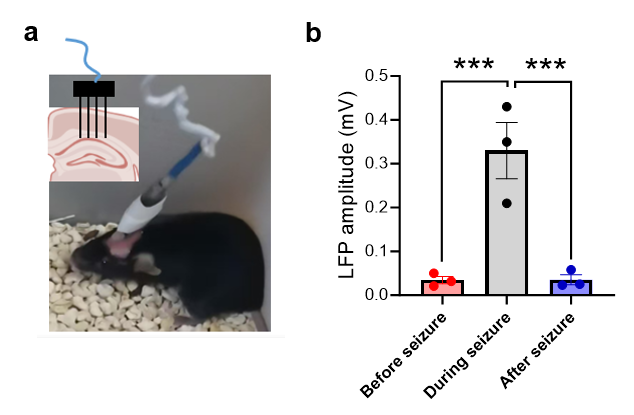


**Figure S2.** **In vivo electrophysiological recording after KA local injection. a** In Sixteen-month-old aged WT mice, kainic acid (KA, 500 nL, 0.5 μg/μL) was locally injected into the hippocampal CA1 subset, and a multi - channel electrode was implanted to 0.1 mm above the injection site. Forty minutes after KA injection, the mice appeared seizures behaviors, such as facial twitching, chewing, head nodding and forelimb clonus. **b** Quantitative analysis on the average amplitude of local field potentials (LFP) using electroencephalogram (EEG) data in (**a**). 15 s-long segments of EEG were selected from three specific time periods: 180 s prior to the onset of epileptic seizure behavior (before seizure), during the epileptic seizures (during seizure), and 180 s after epileptic seizures (after seizure), respectively. Repeated measures ANOVA followed by Tukey’s *post-hoc* test, ****P* < 0.001. 3 seizures from 3 mice.


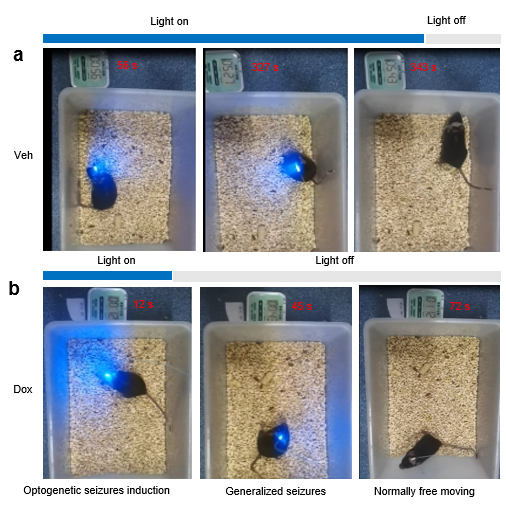


**Figure S3. Aged 16-month-old Tg hTau368 mice treated with Dox for 2 months exhibited shorter latency to optogenetically induced generalized seizures (GS). a** In the Veh group, one mouse required 327 s of optogenetic seizure induction to reach GS. 16 s after the seizure, the mouse resumed normal free movement. **b** In the Dox group, one mouse needed only 45 s of optogenetic seizure induction to reach GS. 27s after the seizure, the mouse returned to normal free - moving behavior.


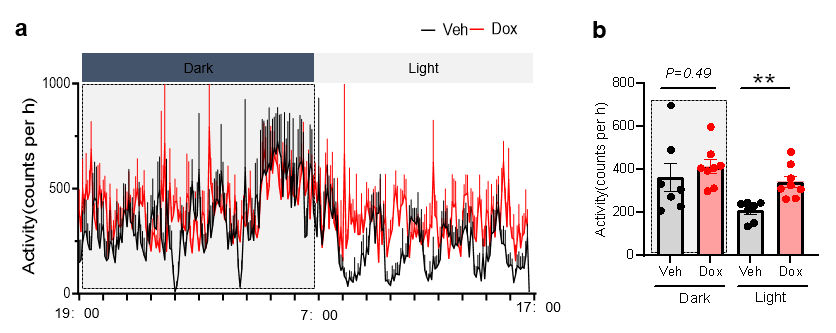


**Figure S4. Increased locomotor activity in aged Tg hTau368 mice. a-b** The number of movements every 5 min at different times within 24 h of aged Tg hTau368 mice treated with Veh/Dox for 2 months (**a**), and (**b**) quantitative statistics of mean movements during night (Dark, 19:00-7:00) and day (Light, 7:00-19:00). Unpaired Student’s t - test was used, with ***P* < 0.01. 16-month - old homozygous mice, 7 - 8 mice per group. Data were presented as mean ± SEM.

**
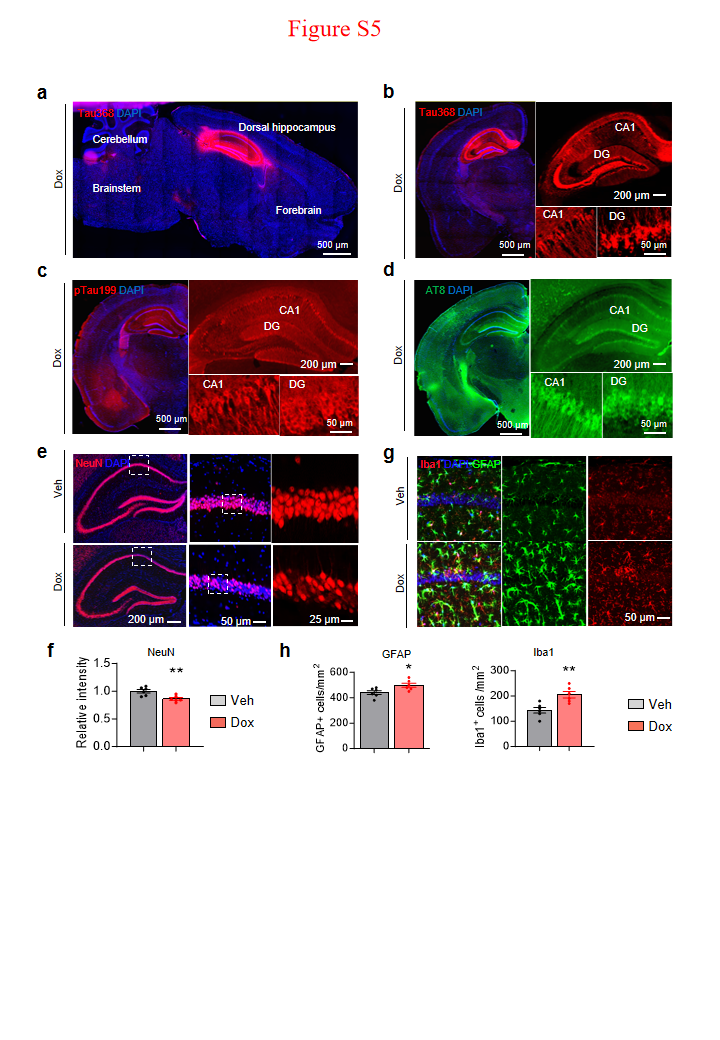
**

**Figure S5. Tau accumulation, neuronal loss and glial activation in the hippocampus of aged Tg hTau368 mice with 2 months of Dox treatment. a, b** Representative sagittal (**a**) and coronal (**b**) immunofluorescence staining images shown relatively prominent hTau368 expression in the hippocampus following 2-month Dox treatment of 16-month homozygous Tg hTau368 mice. **c, d** Accumulation of phosphorylated tau in the hippocampus of aged Tg hTau368 mice. Representative immunofluorescence staining images of brain sections from 16-month Tg hTau368 mice treated with Dox for 2 months, detected by pT199-tau (**c**) and AT8 (**d**) antibodies. **e, f** Neuronal loss in the hippocampus of Dox-administered aged Tg hTau368 mice. (**e**) Representative immunofluorescent images of NeuN-labeled cells in the hippocampus of sixteen-month Tg hTau368 mice following 2 months of Veh and Dox treatment. (**f**) Dox group had lower NeuN intensity in the CA1 pyramidal layer than Veh group. Unpaired Student’s *t*-test, ***P* < 0.01; Data are presented as Mean±SEM, *n* = 6 mice per group. **g, h** Dox-administered 16-month Tg hTau368 mice had increased number of GFAP-labeled astrocyte and Iba1-labeled microglia in the hippocampus. Unpaired Student’s *t*-test, **P* < 0.05, ***P* < 0.01, *n.s., no significant*; Data are presented as mean ± SEM, *n* = 6 mice per group.


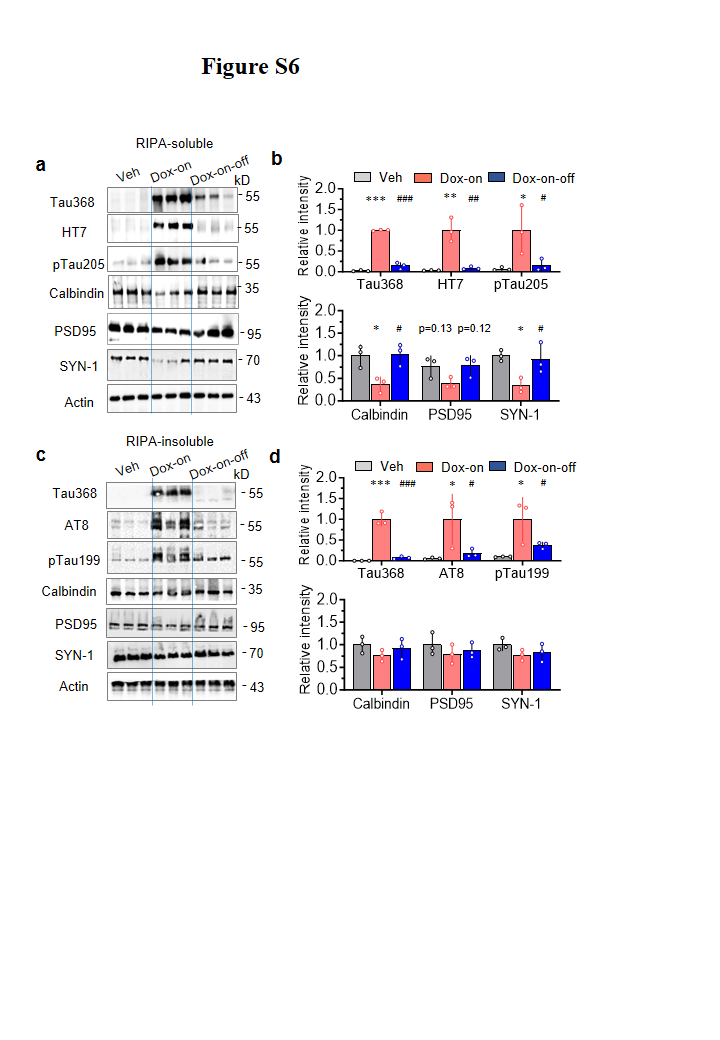


**Figure S6. Hippocampal tau aggregation correlated with reduced CB and synapse-related proteins.** (**a, c**) Representative Western blotting images of RIPA-soluble/RIPA-insoluble lysate of hippocampus and (**b, d**) Quantitative statistics. In Tg hTau368 mice treated with Dox for 2 months (Dox on), hTau368 and pTau aggregation occurred in the hippocampus, and the levels of CB and synapse - related proteins were reduced. However, after withdrawing Dox for 3 months (Dox - on - off), the clearance of hTau368 and pTau was accompanied by the recovery of the expression of CB and synapse - related proteins. One - way analysis of variance was performed, followed by Post hoc Tukey’s multiple comparison test. **P* < 0.05, ***P* < 0.01, ****P* < 0.001 vs Veh group, ^#^*P* < 0.05, ^##^*P* < 0.01, ^###^*P* < 0.001 vs Dox-on group. 7-month-old homozygous hTau368 transgenic mice, 3 mice per group. Data are presented as mean±SEM.


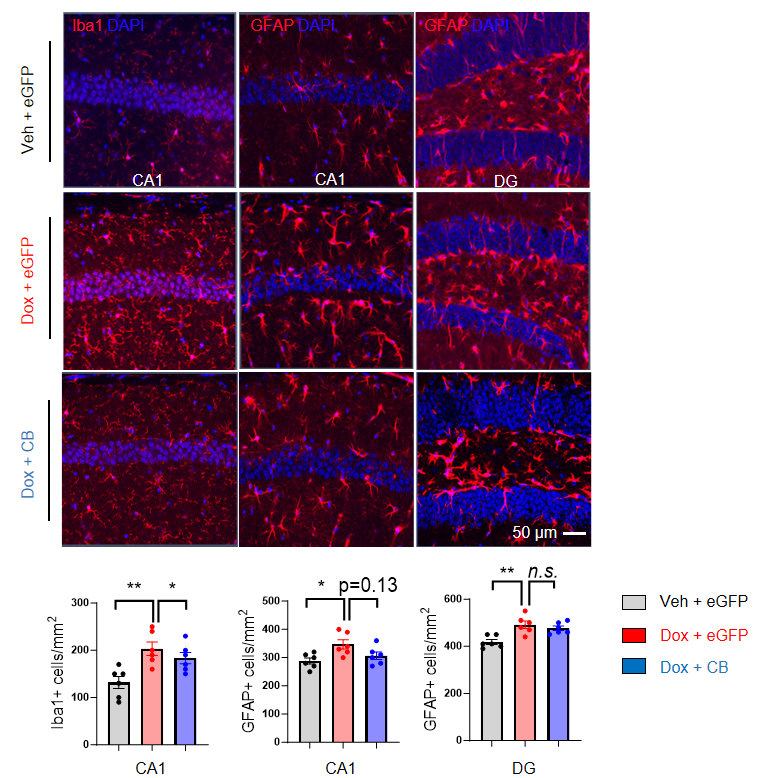


**Figure S7. Overexpression of CB in the hippocampus ameliorates neuroinflammation caused by tauopathology.** Representative immunofluorescence staining images of GFAP-labeled astrocyte and Iba1-labeled microglia in Tg hTau368 mice treated with Veh + AAV-CaMKIIa-eGFP (Veh + eGFP), Dox + AAV-CaMKIIa-eGFP (Dox + eGFP) and Dox + AAV-CaMKIIa-Calb1-eGFP (Dox + CB). One-way analysis of variance was performed, followed by Post hoc Tukey’s multiple comparison test. **P* < 0.05, ***P* < 0.01, *n.s., not significant*. Data are presented as mean±SEM, *n* = 6 mice per group.

**Table S1. Viruses and injection sites** i**n this study.**

| Name | Source | Injection site and dosage | Instruction |
| --- | --- | --- | --- |
| pAAV-CaMKIIa-GCaMp6f-WPRE-hGH polyA | Wuhan Brain VTA Technologies Co., Ltd. | Right dCA1(-1.0 mm AP, +1.8 mm ML, -1.5 mm DV) 500 nL | Calcium signals of excitatory neurons recording |
| pAAV-CaMKIIα-ChR2(H134R)-mCherry | Wuhan Brain VTA Technologies Co., Ltd. | Right dCA1 (-1.0 mm AP, +1.8 mm ML, -1.5 mm DV), 500 nL; right dDG (-1.0 mm AP, +1.8 mm ML, -2.1 mm DV), 500 nL | Specifically activation of the dCA1 and dDG excitatory neurons to achieve optogenetic-induced epileptogenesis |
| pAAV-CaMKIIα-ChR2(H134R)-mCherry | Wuhan Brain VTA Technologies Co., Ltd. | Right vCA1 (-3.2 mm AP, +3.2 mm ML, -4.1 mm DV ) 500 nL; DG (-3.2 mm AP, +3.2 mm ML, -4.8 mm DV) , 500 nL | Specifically activation of the vCA1 and vDG excitatory neurons to achieve optogenetic-induced epileptogenesis |
| pAAV-CaMKIIa-Calb1-eGFP | Obio Technology (Shanghai) Corp., Ltd. | Bilateral dCA1(-1.0 mm AP, +1.8 mm ML, -1.5 mm DV), 500 nL；bilateral dDG (-1.0 mm AP, +1.8 mm ML, -2.1 mm DV), 500 nL | Upregulation of CB in the excitatory neurons of the hippocampus |
| pAAV-CaMKIIa-eGFP | Obio Technology (Shanghai) Corp., Ltd. | BilateraldCA1 (-1.0 mm AP, +1.8 mm ML, -1.5 mm DV), 500 nL; bilateral dDG (-1.0 mm AP, +1.8 mm ML, -2.1 mm DV), 500 nL | As a control |


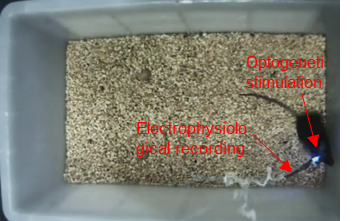


**Screenshot of Video 1.** **Optogenetic induction of seizures and in vivo electrophysiological recording.** This video demonstrates the novel process of optogenetically inducing epileptic seizures in aged mice while synchronously recording brain electrophysiological signals in vivo. At 4 s of the video, we turned on the blue light to activate the hippocampus and induce epileptic seizures. At 4 min and 12 s of the video, the mouse began to exhibit grade 4-5 generalized seizures, and the latency period was recorded as 252 s. After continuing the opto-stimulation for another 15 s, we turned off the blue light and recorded the highest seizure stage. Then, the mouse gradually returned to normal free movement.
